# A NASP (N1/N2)-Related Protein, Sim3, Binds CENP-A and Is Required for Its Deposition at Fission Yeast Centromeres

**DOI:** 10.1016/j.molcel.2007.10.010

**Published:** 2007-12-28

**Authors:** Elaine M. Dunleavy, Alison L. Pidoux, Marie Monet, Carolina Bonilla, William Richardson, Georgina L. Hamilton, Karl Ekwall, Paul J. McLaughlin, Robin C. Allshire

**Affiliations:** 1 Wellcome Trust Centre for Cell Biology and Institute of Cell Biology, School of Biological Sciences, The University of Edinburgh, 6.34 Swann Building, Edinburgh EH9 3JR, Scotland, UK; 2 Institute of Structural and Molecular Biology, School of Biological Sciences, The University of Edinburgh, 6.34 Swann Building, Edinburgh EH9 3JR, Scotland, UK; 3 The Karolinska Institute, Department of Biosciences and Medical Nutrition/University College Sodertorn, Novum 141, 89 Huddinge, Sweden; 4 MRC Human Genetics Unit, Crewe Road, Edinburgh EH4 2XU, Scotland, UK

**Keywords:** DNA

## Abstract

A defining feature of centromeres is the presence of the histone H3 variant CENP-A^Cnp1^. It is not known how CENP-A^Cnp1^ is specifically delivered to, and assembled into, centromeric chromatin. Through a screen for factors involved in kinetochore integrity in fission yeast, we identified Sim3. Sim3 is homologous to known histone binding proteins NASP^Human^ and N1/N2*^Xenopus^* and aligns with Hif1*^S. cerevisiae^*, defining the SHNi-TPR family. Sim3 is distributed throughout the nucleoplasm, yet it associates with CENP-A^Cnp1^ and also binds H3. Cells defective in Sim3 function have reduced levels of CENP-A^Cnp1^ at centromeres (and increased H3) and display chromosome segregation defects. Sim3 is required to allow newly synthesized CENP-A^Cnp1^ to accumulate at centromeres in S and G2 phase-arrested cells in a replication-independent mechanism. We propose that one function of Sim3 is to act as an escort that hands off CENP-A^Cnp1^ to chromatin assembly factors, allowing its incorporation into centromeric chromatin.

## Introduction

The centromere is the chromosomal locus where the kinetochore is assembled to coordinate accurate chromosome segregation. The site of kinetochore assembly is dependent on the deposition of an unusual form of chromatin containing CENP-A. CENP-A (known as CID, HCP-3, Cse4, and Cnp1 in *Drosophila*, *C. elegans*, *S. cerevisiae*, and *S. pombe*, respectively) is a histone H3 variant that replaces H3 in specialized nucleosomes found only at active, but not inactive, centromeres in all eukaryotes (reviewed Cleveland et al., 2003).

Human centromeres are normally found at repetitive arrays of α-satellite DNA and can be assembled de novo on introduced α-satellite DNA (reviewed in Cleveland et al., 2003; Sullivan et al., 2001). However, CENP-A chromatin assembly and propagation are remarkably plastic, as it can assemble and direct kinetochore proteins to assemble on noncentromeric sequences. (Lo et al., 2001; Heun et al., 2006; Castillo et al., 2007). Such observations suggest that assembly of CENP-A chromatin—at regional centromeres composed of arrays of CENP-A nucleosomes—is governed by epigenetic processes. Once assembled at a site, a propagation mechanism must ensure CENP-A chromatin recognition and replenishment during or after each round of DNA replication (Cleveland et al., 2003; Henikoff and Dalal, 2005; Sullivan et al., 2001; Sullivan, 2001). Mechanisms must also operate to ensure that newly made, free CENP-A is delivered only to centromeres for assembly and is excluded from noncentromeric chromatin. This could be achieved by strict control of CENP-A levels; *S. cerevisiae* CENP-A^Cse4^ is regulated by ubiquitin-dependent proteolysis, and overexpression of a nondegradable form results in its broad distribution (Collins et al., 2004). In *Drosophila* and human cells, overexpression of CENP-A allows its assembly at ectopic sites (Heun et al., 2006).

Analyses in metazoan cells indicate that centromeres replicate asynchronously and that CENP-A levels peak in G2 (Shelby et al., 2000; Sullivan and Karpen, 2001). This suggests that there is not, as previously thought, tight coupling between the timing of centromeric DNA replication and CENP-A synthesis and raises the possibility that CENP-A might be deposited after centromere replication, either by replacement of H3 or by filling of chromatin gaps (Furuyama et al., 2006; Shelby et al., 2000; Sullivan, 2001).

Little is known about how CENP-A is delivered to, or assembled on, centromeric DNA and not at other sites in the genome. Human RbAp46/48 and the fission yeast ortholog Mis16 contribute to the localization of CENP-A at centromeres, and RbAp48 can mediate assembly of CENP-A into chromatin in vitro (Furuyama et al., 2006; Hayashi et al., 2004). The human Mis18 complex accumulates at human centromeres between telophase and G1 and is required for the deposition of newly synthesized CENP-A at centromeres. The Mis16/RbAp46/48 and Mis18 proteins appear to regulate the acetylation status of centromeric histones, which in turn affects CENP-A recruitment. Recent analyses indicate that in human cells CENP-A is incorporated in G1 and this requires passage through mitosis (Jansen et al., 2007). Mis18 and associated proteins may prime this by their prior recruitment to centromeres in telophase (Fujita et al., 2007; Maddox et al., 2007). However, neither Mis16 nor Mis18 has been shown to associate with CENP-A. It is important to understand how CENP-A is delivered to and incorporated specifically at centromeres because the assembly of more than one kinetochore on a chromosome can lead to genome instability and chromosomal rearrangements (Sullivan and Willard, 1998).

Analyses in mammalian cells indicate that the histone fold domain is essential for targeting of CENP-A to centromeres (Black et al., 2007; Sullivan et al., 1994). In addition, proteins that associate with human CENP-A—but not H3—nucleosomes have been identified, including two subunits of the FACT chromatin remodeling complex (Foltz et al., 2006; Obuse et al., 2004). However, despite the identification of such CENP-A-associated proteins, little is known about factors such as chaperones that might ensure the safe passage of CENP-A to, or its assembly at, active centromeres and centromeres alone. Recently the *S. cerevisiae* Scm3 protein has been found to be required to assemble a specialized centromeric nucleosome that lacks H2A-H2B (Camahort et al., 2007; Mizuguchi et al., 2007; Stoler et al., 1995). However, it is not known how widespread such an unusual CENP-A nucleosome is in eukaryotes. It is likely that an escort of some type exists for CENP-A because Asf1, CAF1, HIRA, and NASP/N1-N2 act to chaperone H3-H4, with HIRA being specifically required to mediate H3 replacement with H3.3 (reviewed in Loyola and Almouzni, 2004). Moreover, the chaperones NAP1 and Chz1 both bind H2A-H2B and variant H2AZ-H2B dimers; Chz1 shows a preference for H2AZ-H2B, whereas FACT is required for transcription-coupled disassembly of H2A-H2B from nucleosomes (Loyola and Almouzni, 2004; Luk et al., 2007).

**Figure 1:**
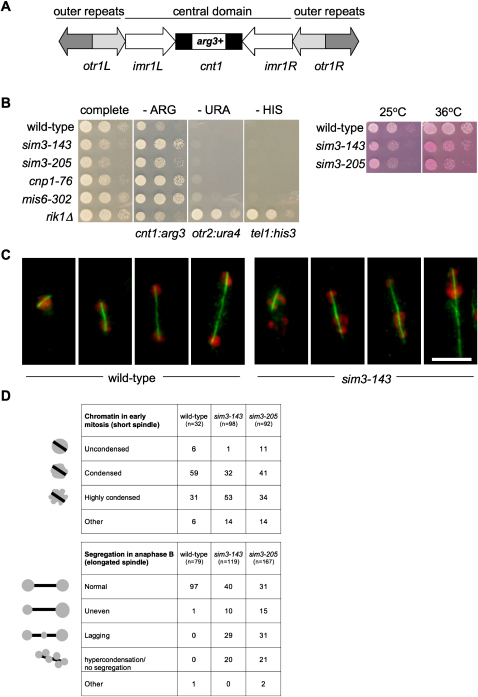
The NASP-Related Protein Sim3 Is Required for Central Core Silencing at Centromeres and Normal Chromosome Segregation (A) *S. pombe* centromeres consist of a central core domain surrounded by outer repeat regions. The *arg3*^+^ insertion at *cnt1* allows monitoring of central core silencing (Pidoux et al., 2003). (B) Left, serial dilution to monitor silencing at central core (*cnt1:arg3*^+^), outer repeat heterochromatin (*otr2:ura4*^+^), and telomeres (*tel1L:his3*^+^), assayed by growth on indicated media. *sim3-143*, *sim3-205*, *cnp1-76*, and *mis6-302* mutants specifically alleviate central core silencing. Outer repeat and telomeric silencing is alleviated in the heterochromatin mutant *rik1*Δ. Right, serial dilution assay to assess growth and viability of *sim3* mutants compared to wild-type at 25°C and 36°C on YES + phloxine B; darker pink colonies contain more dead cells. Strains are FY3027, 6154, 5496, 4462, 5691, and 3606. (C) Chromosome segregation defects in *sim3*-*143*. Wild-type and *sim3*-*143* cells were shifted to 36°C for 6 hr before fixation and immunolocalization with antibodies to α-tubulin (microtubules; green) and DAPI staining (DNA; red). Scale bar, 5 μm. (D) Quantification of chromosome segregation patterns. Numbers are percentages of each pattern in early (short spindles) and late mitosis (elongated spindles) in cultures grown at 36°C (6 hr).

The three centromeres of fission yeast are 35–110 kb and contain two distinct chromatin domains (Figure 1A; reviewed in Pidoux and Allshire, 2004): outer repeat heterochromatin and central domain CENP-A^Cnp1^ chromatin (Partridge et al., 2000; Takahashi et al., 2000). Marker genes inserted within either domain are transcriptionally silenced (Allshire et al., 1994; Allshire et al., 1995). Several proteins have been shown to affect the association of CENP-A with chromatin, including Ams2, Mis6, 15, 16, 17, 18, and Sim4, yet none of these have been reported to bind CENP-A (Chen et al., 2003; Hayashi et al., 2004; Pidoux et al., 2003; Takahashi et al., 2000, 2005). Sim4 and Mis6 form a complex, Mis6 is required for the deposition of newly synthesized CENP-A at centromeres in G2 phase (Pidoux et al., 2003; Takahashi et al., 2000, 2005), and vertebrate Mis6, CENP-I, is also required for the incorporation of newly synthesized CENP-A at centromeres (Okada et al., 2006).

Silencing within the central domain of fission yeast centromeres is dependent on kinetochore integrity (reviewed in Pidoux and Allshire, 2004). Previously we utilized a sensitized marker gene to identify mutants that specifically alleviate central domain silencing (Pidoux et al., 2003). In addition to the kinetochore protein Sim4, we identified mutations in the histone fold domain of CENP-A^Cnp1^. This suggests that silencing within the central domain is dependent on the assembly of CENP-A^Cnp1^ chromatin and that other mutants might identify factors more directly involved in the delivery of CENP-A^Cnp1^ to centromeres and assembly of CENP-A^Cnp1^ chromatin. Here we identify the Sim3 protein, homologous to metazoan histone binding proteins NASP and N1/N2, as being required for kinetochore integrity. Our analyses suggest that one function of Sim3 is to escort CENP-A^Cnp1^ for assembly into the specialized chromatin that underlies the kinetochore.

## Results

### Sim3 Encodes a NASP-Related Protein Required for Central Core Silencing and Normal Chromosome Segregation

The *arg3*^+^ gene inserted within the central core (*cnt1:arg3*^+^) of fission yeast centromere 1 (*cen1*) is transcriptionally silent, resulting in slow growth on plates lacking arginine (−ARG) (Figures 1A and 1B). Two alleles of *sim3* (-*143* and -*205*) that allow faster growth on −ARG plates were identified (Pidoux et al., 2003) (Figure 1B). Sim3 is specifically involved in silencing within the kinetochore domain, as neither allele affects silencing of marker genes placed within the outer repeats (*otr2:ura4*^+^) or adjacent to a telomere (*tel1L:his3*^+^) (Figure 1B). Both *sim3* mutants exhibit impaired growth at all temperatures (Figure 1B). *sim3-143* and *sim3-205* mutants display a variety of abnormal mitotic phenotypes, including hypercondensed chromatin, lagging chromosomes in anaphase, and unequal segregation of chromosomes (Figures 1C and 1D).

**Figure 2:**
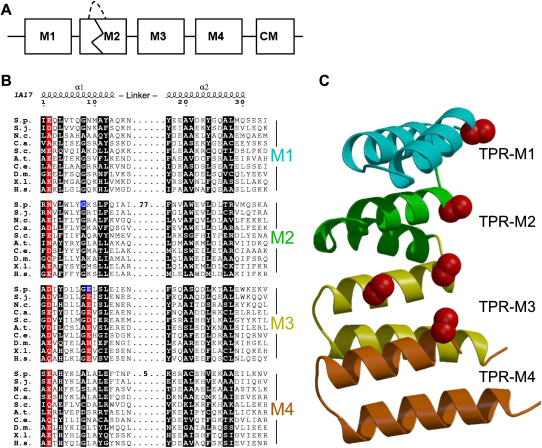
Sim3 Has Interrupted TPR Motifs (A) Motif arrangement in the SHNi-TPR family: four TPR-related motifs (M1–M4) are predicted (see [B]), and a carboxy-terminal motif (CM) also shares similarity. (B) Global alignment of amphipathic helices in motifs –M4 against TPR motif. *S. pombe* Sim3 (S.p.) was aligned with related proteins from other species (see Supplemental Data). The secondary structure from TPR1 of PP5 (PDB code 1A17) is shown above the alignment. Motifs M1, M3, and M4 are predicted to be TPR-like motifs in Human NASP. Global alignment shows that M1, M2, M3, and M4 can be structurally aligned with a TPR motif if insertions are allowed between the amphipathic helices. These insertions are sparse in hydrophobic residues and are likely to be unstructured. The hydrophobic residues that define the TPRs are shown as white-on-black boxes. The positions of mutations in Sim3 are highlighted in blue. Residue 2 of all four SHNi-TPR motifs and residue 9 of M3 are highlighted in red. (C) A model of a four-TPR structure extrapolated from the structure of the three TPRs of PDB code 1fch. Insertions could be accommodated in the links between helices and the links between TPR modules. The Ca and Cb carbons of Residue 2 (M1–M4) and residue 9 of M3 are shown as red spheres. The protein is shown in blue to red graded by sequence number from N to C terminus.

The gene encoding Sim3 was identified by complementation with a high-copy genomic library. Complementing plasmids contained the ORF SPBC577.15c. Sequencing of ORF SPBC577.15c PCR-amplified from *sim3-143* and *sim3-205* cells in multiple mutant progeny from crosses revealed the missense mutations G81E and E207K (Figure 2B), respectively. We conclude that ORF SPBC577.15c encodes the Sim3 protein. Consistent with this, cells lacking the *sim3*^+^ gene are dead at 18°C and display moderate to severe growth impairment at higher temperatures. Furthermore, *sim3*Δ cells display a similar repertoire of phenotypes as *sim3-143* and *sim3-205* cells (Figure S1 available online). This suggests that other unidentified pathways must provide some cover for Sim3 functions.

Sim3 can be structurally aligned with histone binding proteins from fungi to mammals with tetratricopeptide repeats (TPR) (Figure 2). *S. cerevisiae* Hif1 acts as a chaperone for H3/H4 (Ai and Parthun, 2004). N1/N2 is complexed with stored histone H3 and H4 in *Xenopus* oocytes, whereas NASP copurifies with both histone H3 and the H3 replacement variant H3.3 from HeLa cells and has been shown to bind histone H1 (Dilworth et al., 1987; Kleinschmidt and Seiter, 1988; Richardson et al., 2000; Tagami et al., 2004). Although *S. cerevisiae* was reported to lack a NASP-related protein (Aravind et al., 2000), our analyses show that Hif1 aligns with other family members.

The family, which we call SHNi-TPR (*S*im3-*H*if1-*N*ASP *i*nterrupted *T*PR), shares a similar organization: TPR-related motifs M1–M4 and a charged C-terminal motif (Figure 2A). For M1, M3, and M4, at least one SHNi-TPR member has a canonical TPR of 34 residues, whereas other members align to the amphipathic helices in the repeat but have different length unstructured insertions between the two helices (e.g., 77 and 5 residues in M2 and M4 of Sim3; Figure 2B). The global alignment of M1 to M4 shows that large insertions can be accommodated in the linker between the M2 helices. The *sim3-143* and *sim3-205* mutations, G81E and E207K, both occur in the middle of first amphipathic helices of predicted SHNi-TPR repeats (M2 and M3). These bulkier residues, which also change the charge, are expected to destabilize the protein fold. A predicted model is shown in Figure 2C (see Discussion).

### CENP-A^Cnp1^ Levels at Centromeres Are Reduced in *sim3* Mutants

**Figure 3:**
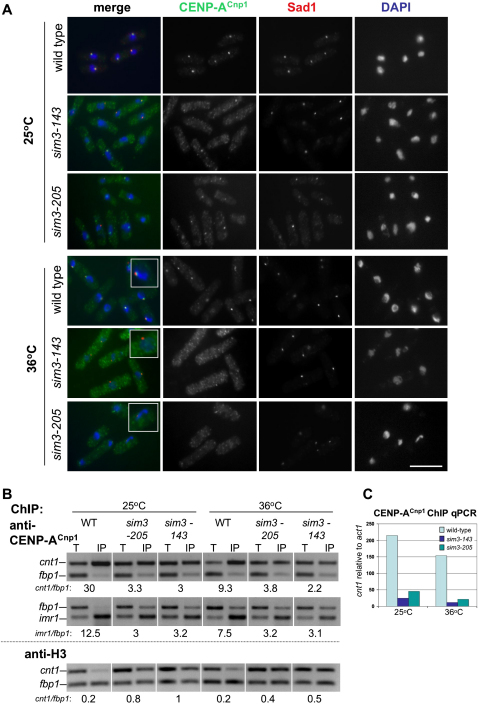
CENP-A^Cnp1^ Levels at Centromeres Are Reduced in *sim3* Mutants (A) CENP-A^Cnp1^ localization in wild-type and *sim3* strains at 25°C and 36°C. Strains were grown at 25°C or shifted to 36°C for 6 hr before fixation and processing for immunolocalization with anti-CENP-A^Cnp1^ (green), anti-Sad1 (red), and DAPI (blue). Scale bar, 10 μm. (B) Top, ChIP of CENP-A^Cnp1^ in wild-type and *sim3* mutants at 25°C and 36°C (6 hr). Immunoprecipitated DNA was analyzed by multiplex PCR. *cnt1* or *imr1* enrichment is measured relative to the *fbp1* euchromatic control and normalized to the input (T, Total) PCR. Bottom, ChIP of histone H3 in wild-type and *sim3* mutants at 25°C and 36°C. The kinetics of fixation differ at 25°C and 36°C, and so ChIPs at the two temperatures are not directly comparable. (C) ChIP of CENP-A^Cnp1^ in wild-type and *sim3* mutants at 25°C and 36°C. In a separate experiment from (B), the level of *cnt1* and *act1* DNA in the input and anti- CENP-A^Cnp1^-immunoprecipitated chromatin was determined by quantitative real-time PCR.

As *sim3* and *cnp1* mutants alleviate silencing at the central kinetochore domain where the CENP-A^Cnp1^ H3 variant chromatin is exclusively assembled, we determined if the level of CENP-A^Cnp1^ at centromeres is altered in *sim3* mutants. Anti-CENP-A^Cnp1^ antiserum was used to localize CENP-A^Cnp1^ in wild-type and mutant cells at 25°C and 36°C. In wild-type G2 fission yeast, all centromeres cluster adjacent to the spindle pole body (SPB) at the nuclear periphery, resulting in a single CENP-A^Cnp1^ signal. However, in *sim3-143* and *sim3-205*, the CENP-A^Cnp1^ signal is reduced or lost, particularly at 36°C, with few cells showing a bright signal adjacent to the SPB (marked by Sad1 staining; Figure 3A). Centromeres remain associated with the SPB in *sim3* mutants, and thus the reduced CENP-A^Cnp1^ localization cannot be explained by loss of centromere-SPB clustering (Figure S2).

The association of CENP-A^Cnp1^ with centromeres was examined by chromatin immunoprecipitation (ChIP; Figure 3B). In agreement with the immunolocalization data, CENP-A^Cnp1^ association with both *cnt* and *imr* regions of the central domain of *cen1* was reduced in both *sim3* mutants (note: ChIPs at 25°C and 36°C are not comparable due to different fixation kinetics). qPCR quantification confirmed loss of centromeric *cnt1* enrichment relative to the euchromatic *act1*^+^ gene in the *sim3* mutants at 25°C and 36°C (Figure 3C). As less CENP-A^Cnp1^ associates with the central domain in *sim3* mutants, it is possible that its place is taken by histone H3 in this defective central domain chromatin. Histone H3 is normally underrepresented in the central domain in wild-type cells, and in *sim3* mutants, concomitant with the observed decrease in CENP-A^Cnp1^, elevated levels of H3 can be detected in the central domain (Figure 3B). This is consistent with the observation that cells with mutant CENP-A^Cnp1^ also have elevated levels of H3 within the central domain and suggests that persistence of H3 may be the default when CENP-A^Cnp1^ chromatin assembly is defective (Castillo et al., 2007). A trivial explanation is that the levels of CENP-A^Cnp1^ are reduced in *sim3* cells, resulting in less CENP-A^Cnp1^ being available for incorporation. The anti-CENP-A^Cnp1^ antiserum is unable to detect CENP-A^Cnp1^ by western; however, the levels of myc-tagged CENP-A^Cnp1^ (expressed from the native promoter as the only source of CENP-A) detected in wild-type, *sim3-143*, and *sim3-205* cells were similar at 25°C and 36°C (Figure S3), as were the levels of H3 (Figure S4).

Previously we have shown that an increased dose of CENP-A^Cnp1^ suppresses *sim3-143* and *sim3-205* phenotypes (Pidoux et al., 2003). Reciprocal to this, overexpression of H3 antagonizes viability of *sim3* mutant cells at 32°C, whereas overexpression of H4 improves viability of both mutants (Figure S5). This again suggests that the defect in central domain chromatin observed in *sim3-143* and -*205* mutants (a high ratio of H3:CENP-A^Cnp1^) is exacerbated by overexpression of histone H3, whereas the provision of more wild-type CENP-A or H4 facilitates CENP-A^Cnp1^ deposition by a defective Sim3 chaperone or by the Sim3-independent pathway implied by *sim3*Δ viability.

These analyses indicate that there is reduced CENP-A^Cnp1^ and increased histone H3 at the central core in cells with defective Sim3. Thus, Sim3 is required to ensure that central domain chromatin is composed mainly of CENP-A^Cnp1^ rather than H3 nucleosomes and suggests that obstructions in the CENP-A^Cnp1^ assembly pathway result in H3 nucleosomes remaining or taking their place. Thus, Sim3 could act to promote CENP-A^Cnp1^ delivery and incorporation in place of H3, or it might act to prevent H3 deposition in the central domain and thereby promote CENP-A^Cnp1^ incorporation instead.

### The Sim3 Protein Is Distributed throughout the Nucleus

**Figure 4:**
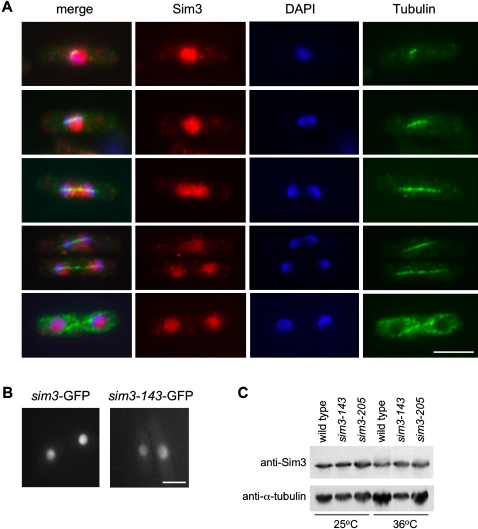
Sim3 Is Distributed throughout the Nucleus (A) Localization of Sim3 to the nucleus throughout the cell cycle. Immunofluorescence of wild-type cells using affinity-purified anti-Sim3 antibody (red), anti-tubulin (microtubules; green), and DAPI (DNA; blue). Merged images are shown on the left. Scale bar, 10 μm. (B) Fluorescence images of live interphase cells expressing Sim3-GFP (FY6326) and Sim3-143-GFP (FY6308). (C) Western analysis using anti-Sim3 antibody to detect levels of Sim3 in wild-type and *sim3* mutants at 25°C and 36°C (6 hr) or anti-tubulin as a loading control.

Other proteins involved in kinetochore function and CENP-A^Cnp1^ association with the central kinetochore domain are themselves located at the central domain. This includes Mis6, 15, 16, 17, and Sim4, though Mis16 is more dispersed over chromatin and Ams2 is only in the nucleus at the onset of S phase and lost in early G2, whereas Mis18 is recruited to centromeres in late mitosis (Chen et al., 2003; Fujita et al., 2007; Hayashi et al., 2004; Pidoux et al., 2003). Anti-Sim3 antibodies were used to localize Sim3 in cells costained with anti-α-tubulin as an indicator of cell-cycle stage. Sim3 localizes throughout the entire nucleus at all cell-cycle stages with no indication of a concentration at centromeres (Figure 4A). In live cells expressing functional GFP-tagged Sim3 (as the only source of Sim3 expressed from the native promoter at the endogenous locus), an even distribution throughout the nucleus was observed. Staining of fixed Sim3-GFP cells with anti-Sim3 or anti-GFP produced a slightly punctate pattern (data not shown), but comparison with the Sim3-GFP pattern in live cells indicates that this is a consequence of fixation (Figure 4B). Costaining with anti-CENP-A^Cnp1^, cell permeabilization and extraction of Sim3-GFP, and ChIP provided no evidence that Sim3 is preferentially localized at centromeres (data not shown), and we conclude that it is a soluble nuclear protein. Although Mis16 and Ams2 are not centromere specific, they have both been shown to be concentrated at centromeres by ChIP (Chen et al., 2003; Hayashi et al., 2004). Western analyses of wild-type and *sim3* mutant extracts from cells grown at either 25°C or 36°C indicate that similar levels of Sim3 protein are present (Figure 4C), suggesting that mutant Sim3 proteins are not labile. In addition, Sim3 localization in cells expressing mutant Sim3-143-GFP or Sim3-GFP appears very similar (Figure 4B).

### CENP-A^Cnp1^ Physically Interacts with Sim3

**Figure 5:**
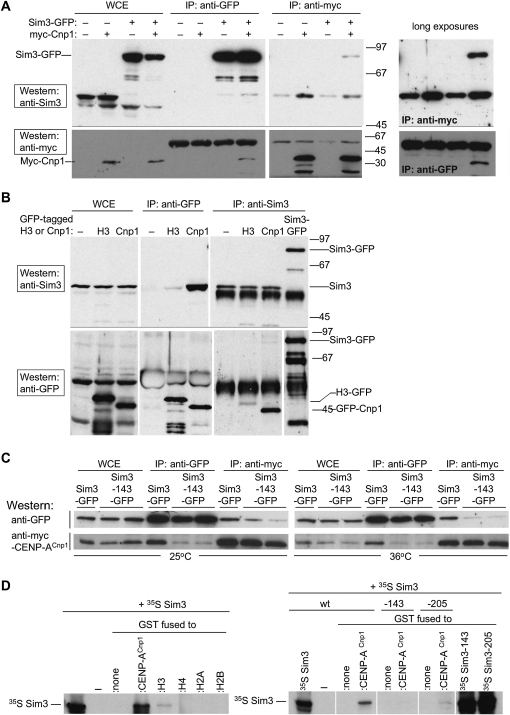
Association of CENP-A^Cnp1^ and H3 with Sim3 (A) Sim3-GFP and myc-CENP-A^Cnp1^ coimmunoprecipitate. Extracts were prepared from cells in which Cnp1 and Sim3 were untagged (FY1645) or which expressed Sim3-GFP (FY6322), or myc-CENP-A^Cnp1^ (FY5927), or both Sim3-GFP and myc-CENP-A^Cnp1^ (FY6374). Immunoprecipitations were performed with sheep anti-GFP or mouse anti-myc (9E10). Immunoprecipitates (IP) and whole-cell extracts (WCE) were analyzed by western blot with rabbit anti-GFP or rabbit anti-myc antibodies as indicated. Positions of Sim3-GFP and Myc-CENP-A^Cnp1^ proteins and molecular weight standards are indicated. Long exposures of two panels are shown on the right for comparison. (B) GFP-CENP-A^Cnp1^ and histone H3-GFP coimmunoprecipitate with Sim3. Extracts were prepared from strains expressing no tagged proteins (FY1645), GFP-CENP-A^Cnp1^ (FY5205), histone H3-GFP (FY6443, a gift from Mohan Balasubramanian), or Sim3-GFP (FY6322). Immunoprecipitations were performed with sheep anti-GFP or rabbit anti-Sim3. IPs and WCEs were analyzed by western blot with rabbit anti-Sim3 or rabbit anti-GFP antibodies as indicated. Positions of Sim3, Sim3-GFP, H3-GFP, and GFP-CENP-A^Cnp1^ proteins and molecular weight standards are indicated. Different length exposures are shown for different panels to allow relevant bands to be seen clearly. (C) Reciprocal coimmunoprecipitations from extracts of cells expressing Sim3-GFP and myc-CENP-A^Cnp1^ (FY6374) or Sim3-143-GFP and myc-CENP-A^Cnp1^ (FY6368/9), grown at 25°C or 36°C (6 hr). Antibodies as in (A). (D) Sim3 and CENP-A^Cnp1^ interact in vitro. Left, ^35^S-labeled Sim3 produced by in vitro transcription and translation was incubated with GST fusion proteins, and pull downs were analyzed by SDS-PAGE and fluorography. ^35^S-labeled Sim3 (1/5th) was run in the left lane. Right, binding of mutant ^35^S-labeled Sim3-143 and Sim3-205 to GST-CENP-A^Cnp1^ was compared to wild-type in the in vitro binding assay.

Sim3 is distributed throughout the nucleus, affects CENP-A^Cnp1^ levels at centromeres, and has a similar domain organization as proteins known to bind histones. Thus, although Sim3 is not concentrated at centromeres, one role for it might be to escort CENP-A^Cnp1^ to the central domain of centromeres and therefore Sim3 would be expected to interact with CENP-A^Cnp1^. To determine if Sim3 is associated with CENP-A^Cnp1^, immmunoprecipitations were performed with strains containing different combinations of tagged Sim3 and CENP-A^Cnp1^ (Figure 5 and Figure S6). myc-CENP-A^Cnp1^ was coimmunoprecipitated with Sim3-GFP only when both tags were present. Reciprocally, Sim3-GFP was coimmunoprecipitated with myc-CENP-A^Cnp1^ (Figure 5A). In addition, in strains overexpressing GFP-CENP-A^Cnp1^, immunoprecipitation with anti-GFP antibody pulls down GFP-CENP-A^Cnp1^ and Sim3, and GFP-CENP-A^Cnp1^ is detected in anti-Sim3 immunoprecipitates (Figure 5B). We also examined whether H3 can associate with Sim3. Attempts to detect H3 or H4 in Sim3 immunoprecipitates with available anti-H3/H4 antisera were mainly unsuccessful (data not shown), and we therefore utilized strains expressing tagged-H3 or H4 to increase sensitivity. Our analyses indicate that HA-tagged CENP-A^Cnp1^, H3, and H4 can coimmunoprecipiate with Sim3 in cells overexpressing these HA-tagged proteins (nmt41x-HA-CENP-A^Cnp1^; inv1-H3-HA; inv1-H4-HA) (Figure S6). To address whether Sim3 has a preference for CENP-A^Cnp1^ or H3, we compared the level of GFP-tagged-CENP-A^Cnp1^ or -H3 that coimmunoprecipitated with Sim3 in cells expressing approximately equivalent levels of either GFP-tagged histone (Figure 5B). These experiments may indicate that more CENP-A^Cnp1^ than H3 can associate in vivo with Sim3. In reciprocal experiments, less Sim3 was detected in anti-H3-GFP than anti-GFP-CENP-A^Cnp1^ immunoprecipitates (Figure 5B). This suggests that Sim3 may have a preference for CENP-A^Cnp1^ over H3, but potential caveats are that the GFP-tagged histones are present at different levels relative to their respective endogenous histones, and the GFP tag may interfere differentially with the binding of these distinct histones to Sim3.

As *sim3* mutants display decreased levels of CENP-A^Cnp1^ at *cnt1*, we investigated if mutant Sim3 protein is defective in its association with CENP-A^Cnp1^. Coimmununoprecipitates from extracts of cells containing Sim3-GFP and myc-CENP-A^Cnp1^ were compared with those from cells in which mutant Sim3 was tagged with GFP (i.e., Sim3-143-GFP and myc-CENP-A^Cnp1^). Although similar levels of both myc-CENP-A^Cnp1^ and GFP-tagged protein were seen in wild-type versus mutant extracts, reciprocal coimmunoprecipitations indicated that lower amounts of the proteins were together in a complex in *sim3* mutants compared to wild-type (Figure 5C). Thus, CENP-A^Cnp1^ can exist in a complex with Sim3, and this association is reduced in strains with G81E and E207K mutations in the SHNi-TPR repeats of Sim3.

We addressed whether Sim3 directly interacts with CENP-A^Cnp1^ and/or H3. Initially we assessed the ability of GST-Sim3 to pull down recombinant H3-H4-H2A-H2B octamers, H3-H4 tetramers, or soluble histones and found that Sim3 has affinity for histones (Figure S7). In addition, yeast two-hybrid assays indicated that Sim3 interacts with both CENP-A^Cnp1^ and H3 in one configuration (Figure S7). Thus, Sim3 may chaperone both H3 and CENP-A^Cnp1^ in different contexts. We next performed in vitro binding experiments (Figure 5D). ^35^S-labeled Sim3 produced by in vitro transcription-translation was tested for binding to GST or GST fused to CENP-A^Cnp1^, H2A, H2B, H3, and H4. It is possible that a protein in the in vitro transcription-translation reaction mediates the interaction observed; however, substantially more Sim3 associated with GST-CENP-A^Cnp1^ compared with GST-H3 and little or no Sim3 associates with GST, GST-H2A, -H2B, or -H4. Binding of ^35^S-labeled mutant proteins Sim3-143 and Sim3-205 to GST-CENP-A^Cnp1^ was dramatically reduced compared with wild-type Sim3. This is consistent with an interaction between Sim3 and CENP-A^Cnp1^ that is compromised by altered residues in M2 and M3 of the SHNi-TPRs (Figure 2C).

The findings that Sim3 binds CENP-A^Cnp1^ in vitro and may associate preferentially with CENP-A^Cnp1^ relative to H3 in cell extracts are consistent with Sim3 acting as an escort for CENP-A^Cnp1^. By delivering CENP-A^Cnp1^ to putative chromatin assembly factors, Sim3 would ensure that CENP-A is incorporated into the centromeric chromatin that underlies the kinetochore. This does not exclude the possibility that Sim3 also acts to chaperone histone H3.

### Sim3 Is Required for the Incorporation of Newly Synthesized CENP-A^Cnp1^ at Centromeres

**Figure 6:**
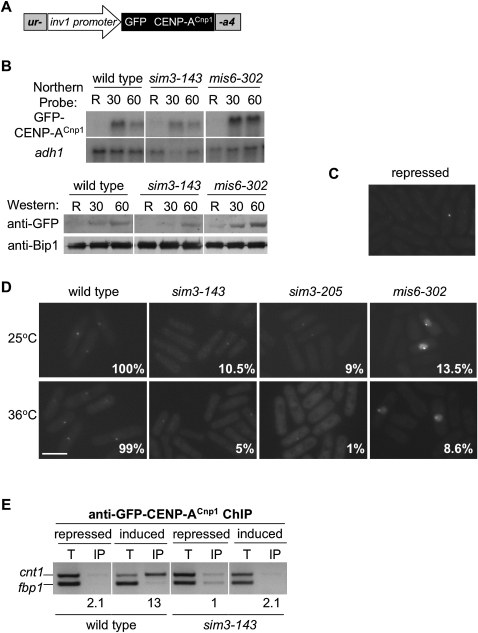
Sim3 Mediates the Incorporation of Newly Synthesized GFP-CENP-A^Cnp1^ at Centromeres (A) Schematic of strain with inducible GFP-CENP-A^Cnp1^ expressed from *inv1* promoter. (B) Northern and western time-course analysis showing induction of GFP-CENP-A^Cnp1^ transcript and protein from the invertase promoter (*inv1*) in wild-type (FY8481), *sim3-143* (FY8482), and *mis6-302* (FY8519) at 25°C. *adh1* was used as a loading control for northern analysis. Levels of induced GFP-CENP-A^Cnp1^ protein were determined by anti-GFP western analysis, and Bip1 was used as a loading control. R, repressed conditions; times of induction are given in minutes. (C) Cells grown in repressed conditions (10% glucose) at 25°C were fixed and analyzed by fluorescence microscopy; very few cells showed a GFP-CENP-A^Cnp1^ signal (<5%). (D) Incorporation of newly synthesized GFP-CENP-A^Cnp1^ in wild-type, *sim3-143*, *sim3-205*, and *mis6-302* at 25°C and 36°C. Cultures were grown at 25°C or shifted to 36°C for 5 hr under repressed conditions, then *inv1*-GFP-CENP-A^Cnp1^ was induced by switching to sucrose media for a further hour at either 25°C or 36°C (for details see text). Cells were fixed and analyzed by fluorescence microscopy, and the presence of the characteristic CENP-A^Cnp1^ signal was scored for each strain and condition (percentage indicated; n = 200). (E) Anti-GFP ChIP on wild-type and *sim3-143* strains containing *inv1*-GFP-CENP-A^Cnp1^ at 25°C, grown under repressed or induced (1 hr) conditions. ChIPs were analyzed by PCR: *cnt1* enrichment is measured relative to the *fbp1* euchromatic control and normalized to the input.

It has been proposed that CENP-A^Cnp1^ is deposited by both replication-coupled and replication-independent mechanisms (Takahashi et al., 2005). To further investigate CENP-A^Cnp1^ deposition at different phases of the cell cycle and to address the involvement of Sim3 in these processes, we set up a system to rapidly induce GFP-CENP-A^Cnp1^. A GFP-CENP-A^Cnp1^ construct under the control of the invertase (*inv1*) promoter was introduced into wild-type (*cnp1*^+^) cells (Figure 6A). The *inv1* promoter has the advantage of being induced within 30–90 min of switching from glucose to sucrose-rich medium and can be induced during cell-cycle arrests (Iacovoni et al., 1999). Northern and western analyses indicate that the GFP-CENP-A^Cnp1^ transcript and protein are induced, reaching maximum levels within 60 min after switching to sucrose (Figure 6B). Analysis by fluorescence microscopy indicated that very few cells (<5%) showed a GFP-CENP-A^Cnp1^ signal under repressed conditions (glucose) (Figure 6C). Upon induction of *inv1*-GFP-CENP-A^Cnp1^ for 60 min, all cells contained the characteristic CENP-A^Cnp1^ spot (Figure 6D). Staining with an anti-Sad1 confirmed that this signal is adjacent to the SPB and thus represents centromeres (Figure S8).

To determine whether Sim3 is required for the incorporation of newly synthesized CENP-A^Cnp1^ into centromeric chromatin, expression of *inv1*-GFP-CENP-A^Cnp1^ was induced in wild-type, *sim3-143*, and *sim3-205* cells at 25°C and 36°C (Figure 6D). After 60 min induction at 25°C, we consistently observed a GFP dot in all wild-type nuclei. In contrast, less than 11% of *sim3-143* or *sim3-205* cells displayed a GFP-CENP-A^Cnp1^ signal, which was substantially weaker than that seen in wild-type cells. Northern and western analyses indicate that GFP-CENP-A^Cnp1^ is produced in *sim3* mutants (Figure 6B). For analyses at 36°C, cells were shifted to 36°C for 5 hr, prior to induction of *inv1*-GFP-CENP-A at 36°C for an additional hour. Most (99%) of the wild-type cells exhibited a strong GFP-CENP-A^Cnp1^ dot in the nucleus after induction, but less than 5% of *sim3* cells exhibited GFP-CENP-A^Cnp1^ at centromeres. As a control, the incorporation of GFP-CENP-A^Cnp1^ was monitored in a *mis6-302* mutant, which has previously been shown to be defective in incorporation of CENP-A^Cnp1^-GFP induced from the *nmt1* promoter (Takahashi et al., 2000). As expected, no GFP-CENP-A^Cnp1^ centromeric signal was observed in most *mis6-302* cells at 25°C or 36°C (Figure 6D). However, in a proportion of *mis6-302* cells (13.5% and 8.5%), a very strong signal was observed at centromeres and GFP-CENP-A^Cnp1^ appeared to fill the nucleus. This confirms that Mis6 is required for the deposition of new CENP-A^Cnp1^ at centromeres in most cells. It is not known why GFP-CENP-A^Cnp1^ overaccumulates in a proportion of *mis6-302* cells.

Anti-GFP ChIP was performed on wild-type and *sim3-143* cells before and after induction of GFP-CENP-A^Cnp1^ at 25°C. In wild-type cells, little GFP-CENP-A^Cnp1^ was found to associate with *cnt1* prior to induction, but after 60 min induction, robust enrichment of *cnt1* relative to *fbp1* was observed (Figure 6E). In *sim3-143* cells, little GFP-CENP-A^Cnp1^ associates with *cnt1* after induction. These analyses indicate that, in wild-type cells, newly made GFP-CENP-A^Cnp1^ is efficiently deposited in the central domain chromatin. However, although GFP-CENP-A^Cnp1^ is induced, it is inefficiently incorporated at centromeres in *sim3* mutant cells. This demonstrates that one function of Sim3 is to allow newly synthesized CENP-A^Cnp1^ to be incorporated into centromeric chromatin.

The localization of induced *inv1*-HA-tagged histone H3 to chromatin was unaffected in *sim3* mutants and was identical to that observed in wild-type cells, therefore *sim-143* and *sim3-205* do not appear to affect the deposition of new histone H3 in this relatively crude assay (Figure S9). Additionally, the fact that H3 is detected in the central domain in place of CENP-A in *sim3* mutants suggests that the ability to assemble H3 into chromatin is not compromised in *sim3* mutants (Figure 3). If Sim3 plays a major role in the deposition of H3, cells expressing defective Sim3 would be expected to have a general defect in chromatin integrity, which might affect gene expression. It is also possible that the mutations in *sim3* indirectly affect centromeres through changes in expression of centromere components. To address these concerns, expression profiling of *sim3-143* and *sim3-205* cells was performed (see Table S1). mRNA levels in logarithmically growing cultures of *sim3* mutant cells were compared to those in wild-type. Lists of misregulated genes in *sim3* mutants relative to wild-type were established. Using a standard 2-fold cutoff for changes in gene expression, only 20 genes were >2-fold up- or downregulated by the stronger *sim3-205* allele and no genes were affected by the weaker *sim3-143* allele. Using a less stringent 1.5-fold cutoff, 43 genes were affected by *sim3-143* and 113 genes were affected by *sim3-205*. Surprisingly, only one gene (SPAC1F8.04c) was similarly affected by both *sim3* alleles. Thus, we conclude that relatively few genes and distinct sets of genes were affected in the two *sim3* mutant alleles. The annotation of the affected genes indicates that no gene products known to be involved in centromere function are affected, thus arguing against indirect effects of *sim3* on CENP-A chromatin assembly. The fact that so few genes are affected in *sim3* mutants indicates that Sim3 is not required for general chromatin integrity and suggests that one of its main functions is to escort CENP-A^Cnp1^. However, other roles for Sim3 in H3 chromatin integrity may remain to be uncovered.

### Sim3 Is Required to Aid the Deposition of CENP-A^Cnp1^ during S and G2

During S phase, newly synthesized histones are deposited on DNA by a chromatin assembly process that is tightly coupled to DNA synthesis at the replication fork. However, replacement histones such as H3.3 and H2AZ are deposited in a replication-independent manner and require distinct assembly factors (Mizuguchi et al., 2004; Tagami et al., 2004).

Human CENP-A is synthesized in G2, and its incorporation at centromeres is not synchronized with their replication (Shelby et al., 2000; Sullivan and Karpen, 2001). CENP-A^Cnp1^-GFP is also incorporated at centromeres in fission cells blocked in G2 (Takahashi et al., 2005). As ∼70% of cells in an asynchronous culture are in G2, the data presented above (Figure 6) suggest that, like the Mis6 (control), Sim3 participates in a CENP-A^Cnp1^ chromatin assembly process in interphase. However, to test more rigorously if new CENP-A^Cnp1^ produced from *inv1*-GFP-CENP-A^Cnp1^ is incorporated during G2 or other cell-cycle stages, this construct was combined with the temperature-sensitive *cdc10* (G1 phase arrest), *cdc25* (G2 phase arrest) mutations, or analyzed cells in the presence of hydroxyurea (HU; S phase arrest).

**Figure 7:**
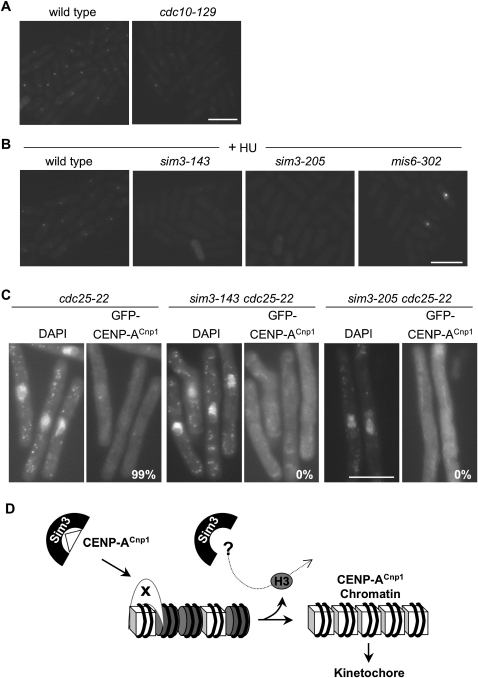
Sim3 Aids the Deposition of GFP-CENP-A^Cnp1^ during S and G2 (A) Wild-type and *cdc10-129* mutant (G1 arrest) cells were incubated at 36°C for 3 hr followed by induction of *inv1*-GFP-CENP-A^Cnp1^ for 60 min in sucrose medium (4 hr total at 36°C). Cells were fixed and analyzed for presence of GFP-CENP-A^Cnp1^ spot by fluorescence microscopy. (B) Wild-type, *sim3-143*, *sim3-205*, and *mis6-302* cells were arrested in S phase by the addition of 25 mM hydroxyurea (HU) for 4 hr at 25°C, followed by induction of *inv1*-GFP-CENP-A^Cnp1^ in sucrose media containing HU for a further 1 hr (5 hr total). Cells were analyzed as in (A). (C) Incorporation of newly synthesized GFP-CENP-A^Cnp1^ in *cdc25-22* (FY8518), *sim3-143 cdc25-*22 (FY8717), and sim3*-205 cdc25-22* (FY8718) strains at 36°C. Cultures grown at 25°C were shifted to 36°C for 3 hr in repressed conditions, then *inv1*-GFP-CENP-A^Cnp1^ was induced for a further 1 hr at 36°C. Cells were fixed, DAPI stained, and analyzed by fluorescence microscopy for the presence of GFP-CENP-A^Cnp1^ signal (n = 200 for each strain). Scale bar, 10 μm. (D) Model: Sim3 (C shape) acts a classic chaperone, directly binding CENP-A^Cnp1^ (white triangle), escorting it to the centromere, and handing it over to centromere-associated chromatin assembly factors (white crescent, X) that incorporate CENP-A^Cnp1^ in place of histone H3. CENP-A^Cnp1^ and H3 nucleosomes are shown as white cubes and gray cylinders, respectively. Evicted H3 may also be received by Sim3.

Cdc10 is required for the initiation of S phase; after 4 hr at 36°C, *cdc10-129* cells are elongated, indicative of a G1/S cell-cycle arrest (Forsburg and Nurse, 1991). *cdc10-129* cells were incubated at 36°C for 3 hr and then *inv1*-GFP-CENP-A^Cnp1^ was induced by switching to sucrose for an additional 60 min incubation at 36°C (4 hr total). Only weak GFP-CENP-A^Cnp1^ foci were visible in a few cells in *cdc10-129*-arrested cells (Figure 7A), suggesting that newly produced CENP-A^Cnp1^ is not efficiently incorporated at centromeres in cells arrested in G1. Thus the CENP-A^Cnp1^ assembly pathway may be downregulated in G1.

To determine whether Sim3-dependent CENP-A^Cnp1^ deposition operates during S phase, wild-type, *sim3-143*, and *sim3-205* cells were arrested in early S phase by the addition of HU. After 4 hr in HU, new GFP-CENP-A^Cnp1^ was induced (1 hr in HU + sucrose), and GFP-CENP-A^Cnp1^ foci were clearly formed in wild-type, but not *sim3* or most *mis6* (control), cells (Figure 7B). Thus CENP-A^Cnp1^ can be deposited in S phase without ongoing replication.

Cdc25 is required to activate Cdc2 cyclin-dependent kinase and allow cells to enter mitosis (Forsburg and Nurse, 1991). After 4 hr at 36°C, *cdc25-22* cells are blocked at G2/M as indicated by elongated cell morphology. After 3 hr incubation at 36°C, *inv1*-GFP-CENP-A^Cnp1^ was induced in *cdc25-22* cells for 60 min at 36°C. All *cdc25-22* cells displayed a GFP-CENP-A^Cnp1^ dot (Figure 7C), indicating that GFP-CENP-A^Cnp1^ produced in G2-arrested cells is deposited at centromeres. However, in parallel experiments, a centromeric GFP-CENP-A^Cnp1^ signal was not detectable in *sim3-143 cdc25-22* and *sim3-205 cdc25-22* cells. This indicates that the NASP-related protein Sim3 is required for the efficient replication-independent deposition of CENP-A^Cnp1^ at fission yeast centromeres during G2.

Thus, Sim3 is required in both S- and G2-arrested cells to allow CENP-A^Cnp1^ incorporation at centromeres. Although distinct mechanisms of CENP-A^Cnp1^ deposition have been found to operate during S and G2 phases of the cell cycle (Takahashi et al., 2000, 2005), it appears that Sim3 is required to escort nascent CENP-A^Cnp1^ in both S and G2 phase to CENP-A^Cnp1^ chromatin assembly factors for incorporation at centromeres (Figure 7D).

## Discussion

The *sim3* mutants were identified through their alleviation of silencing within the central kinetochore domain of fission yeast centromeres. Here, we have shown that the *sim3* mutations reside in an ORF that can be aligned with the known histone binding proteins N1/N2, NASP, and Hif1. We have demonstrated that Sim3 is required for normal levels of CENP-A^Cnp1^ at the central domain of centromeres and probably interacts directly with CENP-A^Cnp1^ in vitro. In addition, our analyses indicate that Sim3 can associate with H3, suggesting that Sim3 may have other roles in chromatin assembly and integrity. However, expression profiling indicates that Sim3 does not act generally to maintain chromatin. In addition, immunoprecipitations from fission yeast extracts suggest that Sim3 may preferentially associate with CENP-A^Cnp1^ compared to histone H3.

The two *sim3* mutants isolated have altered residues in the conserved interrupted TPR-like repeats (SHNi-TPR), and both disrupt Sim3-CENP-A^Cnp1^ complex formation in vitro and in vivo. The Sim3 protein is not a kinetochore protein but is distributed throughout the nucleus. Cells with defective Sim3 are unable to incorporate newly synthesized CENP-A^Cnp1^ at centromeres in S or G2 phases. Together, these data are consistent with a model in which Sim3 acts as an escort for CENP-A^Cnp1^, ensuring that it is delivered to centromeres. We suggest that Sim3 hands off CENP-A^Cnp1^ to other assembly factors located at centromeres (Figure 7D). In this way, Sim3 might also contribute to the specificity of incorporation and prevent the inappropriate assembly of CENP-A^Cnp1^ into noncentromeric chromatin. Centromere-associated proteins such as Ams2, Mis6, 15, 16, 17, 18, and Sim4 are known to affect CENP-A^Cnp1^ incorporation in the central domain and are candidates for the putative CENP-A^Cnp1^ acceptors. However, to date, none of these kinetochore proteins have been shown to associate with CENP-A^Cnp1^. Indeed, to the best of our knowledge, Sim3 is the first protein in fission yeast, which has regional rather than point centromeres, that has been shown to associate with CENP-A^Cnp1^. Hence, Sim3 may contribute to the propagation of CENP-A^Cnp1^ chromatin at a specific locus by only surrendering CENP-A^Cnp1^ to factors exclusively associated with active centromeres. Such factors might include the ortholog of *S. cerevisiae* Scm3 (Camahort et al., 2007; Mizuguchi et al., 2007; Stoler et al., 2007), but this remains to be tested.

It is well established that canonical histone H3 can be evicted and replaced by the H3.3 variant in metazoan cells. This provides the paradigm for replication-independent histone replacement (reviewed in Henikoff and Ahmad, 2005). The replacement of H3 by H3.3 is induced by transcription and, because it can occur in interphase cells, is uncoupled from replication. H3.3 complexes with the chaperones HIRA and ASF1A, which are required to allow its assembly into chromatin—interestingly, NASP also associated with H3.3 (Tagami et al., 2004). The Swr1 complex promotes the replacement of core histone H2A with the variant H2AZ, and its Swc2 subunit directly binds H2AZ and is required for H2A-H2AZ exchange (Mizuguchi et al., 2004; Wu et al., 2005). Chz1 passes H2AZ to Swr1 but does not participate in H2AZ-H2A replacement (Luk et al., 2007). Our analyses detected elevated levels of histone H3 in the central kinetochore domain in cells with defective Sim3. One explanation is that histone H3 is deposited at centromeres by default during replication and is subsequently evicted and replaced with the kinetochore-specific H3 variant CENP-A^Cnp1^ in a manner similar to that described for replacement of H3 by H3.3 during transcription (Ahmad and Henikoff, 2002; McKittrick et al., 2004; Mito et al., 2005). Such a process may be reliant on a priming step mediated by Mis18 in late metaphase (Fujita et al., 2007; Maddox et al., 2007). Indeed, the detected Sim3-H3 association might be indicative of Sim3 being required to receive evicted H3 after an H3-to-CENP-A exchange event. A complete understanding of such CENP-A histone replacement/exchange factors awaits further investigation. Regardless of the exact CENP-A assembly mechanism, we envisage that fission yeast Sim3 acts ahead of such chromatin assembly or histone exchange factors as a classic chaperone, ensuring that CENP-A^Cnp1^ is handed over to centromere-associated CENP-A assembly factors, but unlike *S. cerevisiae* Scm3, it is not a component of the final structure itself.

As newly synthesized CENP-A^Cnp1^ is deposited at centromeres in both G2- and S phase-arrested cells, CENP-A^Cnp1^ deposition does not appear to be mandatorily coupled to ongoing replication, even during S phase. As CENP-A^Cnp1^ levels decline and histone H3 levels increase at centromeres in *sim3* mutants, it is possible that H3 is initially deposited in S phase and subsequently replaced by nascent CENP-A^Cnp1^ during S and G2. Another possibility is that by binding to CENP-A^Cnp1^ Sim3 acts to prevent its promiscuous incorporation into noncentromeric chromatin. The order of events, players, and specific interactions in such a complex exchange or remodeling event may differ in details between species so that in some organisms CENP-A may be incorporated in G1 after priming in late mitosis (Fujita et al., 2007; Jansen et al., 2007), whereas in other organisms, related events may occur at different cell-cycle stages.

The structural alignment indicates that Sim3 and other SHNi-TPR family members contain a reiterated sequence motif that is an interrupted form of TPR repeat (Figure 2). TPR motifs are normally found in tandem arrays, and structures show that these helical hairpins form a head-to-tail zigzag structure to create a convex face and a concave surface inside the superhelix (reviewed in D'Andrea and Regan, 2003). In some structures, a peptide is shown to bind within the cavity formed by the TPR motifs (Scheufler et al., 2000). In the SHNi-TPR family, position 2 of each repeat has a negatively charged side chain or an amidated version. Moreover, position 9 of M3 is often negatively charged. Intriguingly, these residues line the concave face in a model of four SHNi-TPR repeats and may form an ion binding site or a recognition site for a positively charged section of proteins such as histones (Figure 2C). The mutations identified in Sim3, which affect both in vitro and in vivo interactions with CENP-A^Cnp1^, are predicted to disrupt this putative recognition site.

Thus, Sim3 is related to NASP that has been shown to copurify with both H3 and H3.3 from mammalian cells (Tagami et al., 2004). Curiously, *S. cerevisiae* was reported to lack a NASP protein (Aravind et al., 2000). This might reflect the fact that *S. cerevisiae* kinetochores contain a single CENP-A nucleosome, whereas centromeres in other organisms (including the yeast *C. albicans*) have arrays of CENP-A nucleosomes at each centromere (Baum et al., 2006), and in humans, at least, these contain H2A-H2B (Foltz et al., 2006). In fact, although the *S. cerevisiae* protein Hif1 shares only weak similarity to NASP, N1/N2, and Sim3, it can be aligned with them and has been shown to bind H3/H4 and contribute to their deposition.

It is not known if NASP proteins are required for the deposition of histones in metazoa, but NASP associates with H3 and H3.3 in HeLa cells. It is also not known if NASP associates with CENP-A in these cells. Different organisms may put different emphases on particular mechanisms that restrict CENP-A to centromeres; these may include proteolysis and/or targeting (Black et al., 2007; Collins et al., 2004). In fission yeast, the Sim3 NASP-related protein appears to act as a CENP-A^Cnp1^ chaperone but it may also have other unidentified roles in aiding H3 dynamics, as indicated by its association with H3. It will be interesting to determine if other NASP-like proteins are involved in shepherding CENP-A to centromeres.

## Experimental Procedures

### Induction and Analysis of Newly Synthesized GFP-CENP-A^Cnp1^

GFP-CENP-A^Cnp1^ was placed under the control of the *inv1* promoter and integrated at the *ura4* locus (strain FY8481). The *inv1* promoter was repressed in PMG with 10% glucose and induced by switching to PMG with 4% sucrose as the sole carbon source for 1 hr. To block DNA replication, cells were treated with 25 mM HU for 4 hr at 25°C. For FACS analysis (BD FACSCalibur), cells were fixed in 70% ethanol before staining with propidium iodide.

### Other Methods

In vitro binding assays are described in the Supplemental Data. Microscopy and ChIP were performed as previously described (Pidoux et al., 2003). Immunoprecipitations were performed as previously described (Millband and Hardwick, 2002). Modifications and details of antibodies are given in the Supplemental Data.

## Accession Numbers

The Gene Expression omnibus (GEO) submission series for the sim3 microarray data is GSE7560 at http://www.ncbi.nlm.nih.gov/geo/.

## Supplemental Data

Document S1. Supplemental Experimental Procedures, Supplemental References, Nine Figures, and Three Tables
