# Supplementary material for: A NASP (N1/N2)-Related Protein, Sim3, Binds CENP-A and Is Required for Its Deposition at Fission Yeast Centromeres
Source: Mol Cell. 2007 Dec 28;28(6):1029–44. doi: 10.1016/j.molcel.2007.10.010 (PMC2193228; doi:10.1016/j.molcel.2007.10.010)
Supplement: Document S1. Supplemental Experimental Procedures, Supplemental References, Nine Figures, and Three Tables [file mmc1.pdf]

Molecular Cell, Volume *28*

Supplemental Data

A NASP (N1/N2)-Related Protein, Sim3, Binds CENP-A and Is Required for Its Deposition at Fission Yeast Centromeres

Elaine M. Dunleavy, Alison L. Pidoux, Marie Monet, Carolina Bonilla, William Richardson, Georgina L. Hamilton, Karl Ekwall, Paul J. McLaughlin, and Robin C. Allshire

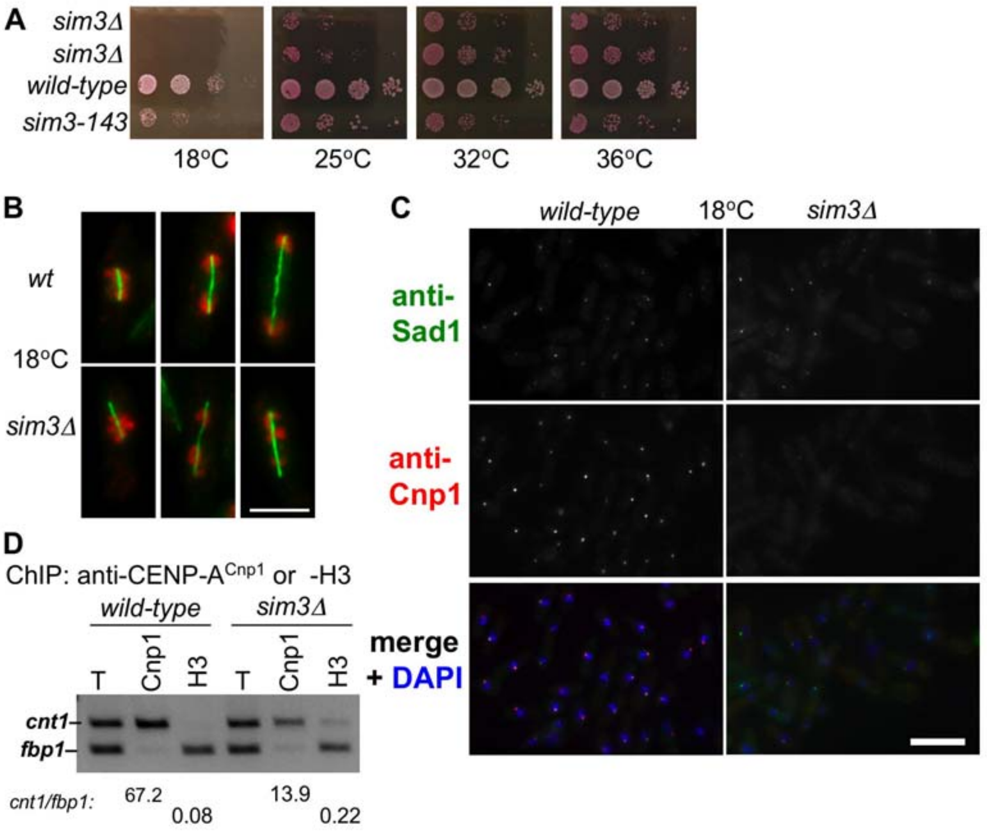

Figure S1

A. *sim3Δ* cells are inviable at 18°C and growth is impaired at 25, 32 and 36°C.
B. Chromosome segregation is defective in *sim3Δ* cells. Representative images of cells stained with anti-tubulin (green) and DAPI (red). Bar = 5 μm.
C. CENP-A^{Cnp1} is lost from centromeres in *sim3Δ* cells. Wild-type and *sim3Δ* cells were grown at 18°C, fixed and stained with anti-Sad1 (SPB marker) and anti-CENP-A^{Cnp1} and DAPI. Anti-Sad1 signal is equivalent in wild-type and *sim3Δ* cells but anti-CENP-A^{Cnp1} signal is significantly reduced in *sim3Δ* cells. Bar = 10 μm.
D. CENP-A^{Cnp1} is reduced and H3 levels increase in the central kinetochore domain of *cen1* of *sim3Δ* cells. Chromatin IP with anti- CENP-A^{Cnp1} and anti-H3 were performed on extracts from wild-type and *sim3Δ* cells. Multiplex PCR was performed with primers specific for *cnt1* of *cen1* and levels compared with a control non-

centromeric locus *fbp1*. The relative amount of *cnt1* product compared to *fbp1* was calculated from band intensities in the IPs and normalised relative to the total input (T). Cells were grown at 32°C and shifted to 25°C for two hours.
Strains: FY11049, 11052.

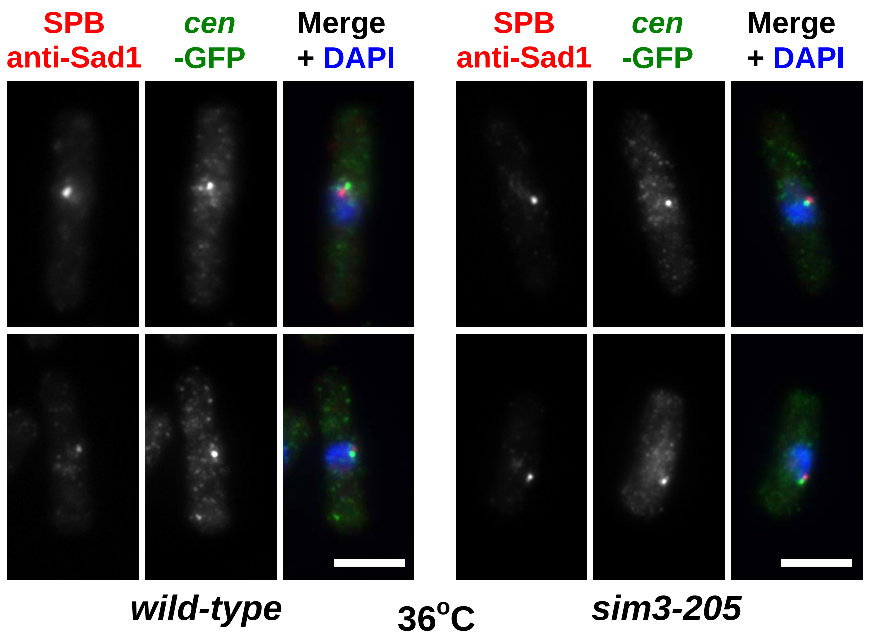

Figure S2

Centromeres remain associated with the SPB in *sim3* mutant cells. Wild-type and *sim3-205* cells bearing lacO/LacI-GFP beacons adjacent to centromeres 1 and 2 were grown at 36°C for 6 hours, fixed and stained with anti-Sad1 (an SPB specific protein) and DAPI. These representative images show that the GFP-beacon is located in close proximity to the SPB in both wild type and *sim3-205* cells. Thus, *sim3-205* does not affect the association of centromere with the SPB in interphase and this supports the conclusion that the loss of CENP-A^{Cnp1} observed by immunolocalisation (Figure 3A) is not simply due to declustering of centromeres. Strains FY10539, 11055.

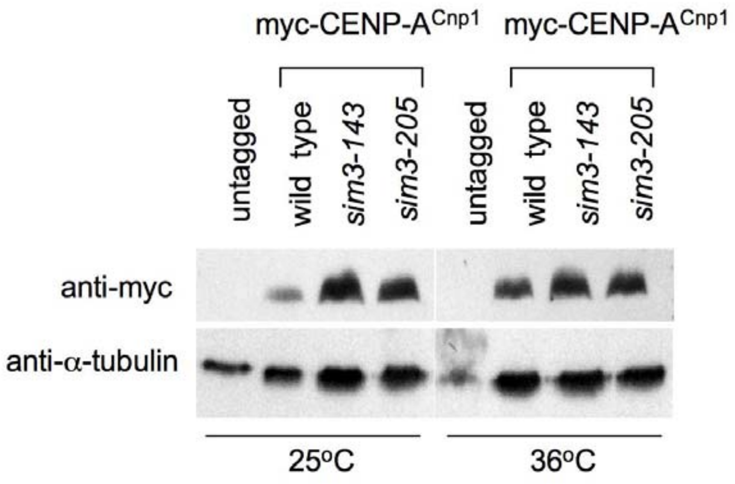

Figure S3

Levels of myc-CENP-A^{Cnp1} protein are similar in wild type and *sim3* mutants grown at 25°C and 36°C (6 hr). Western using anti-myc antibodies to detect myc-tagged CENP-A^{Cnp1} protein (expressed from its endogenous promoter), or anti-tubulin as a loading control.

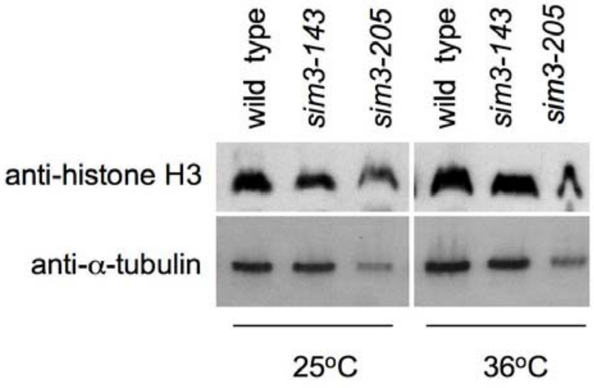

Figure S4

Levels of histone H3 protein are similar in wild type and *sim3* mutants grown at 25°C and 36°C (6 hr). Western analysis using anti-histone H3 C terminal antibody to detect levels of histone H3 in wild type and *sim3* mutants at 25°C and 36°C (6 hr), or anti-tubulin as a loading control.

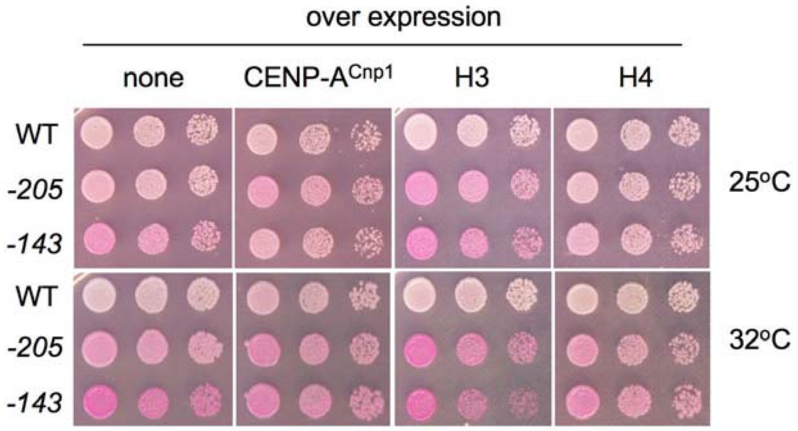

Figure S5

sim3 mutants are partially rescued by increased expression of histone H4 overexpression but antagonised by additional H3.
Serial dilution assay of wild type and *sim3* cells expressing additional CENP-A^{Cnp1}, H3 or H4. Light pink colonies on phloxine B indicate healthy growth; dark pink indicates accumulation of dead cells.

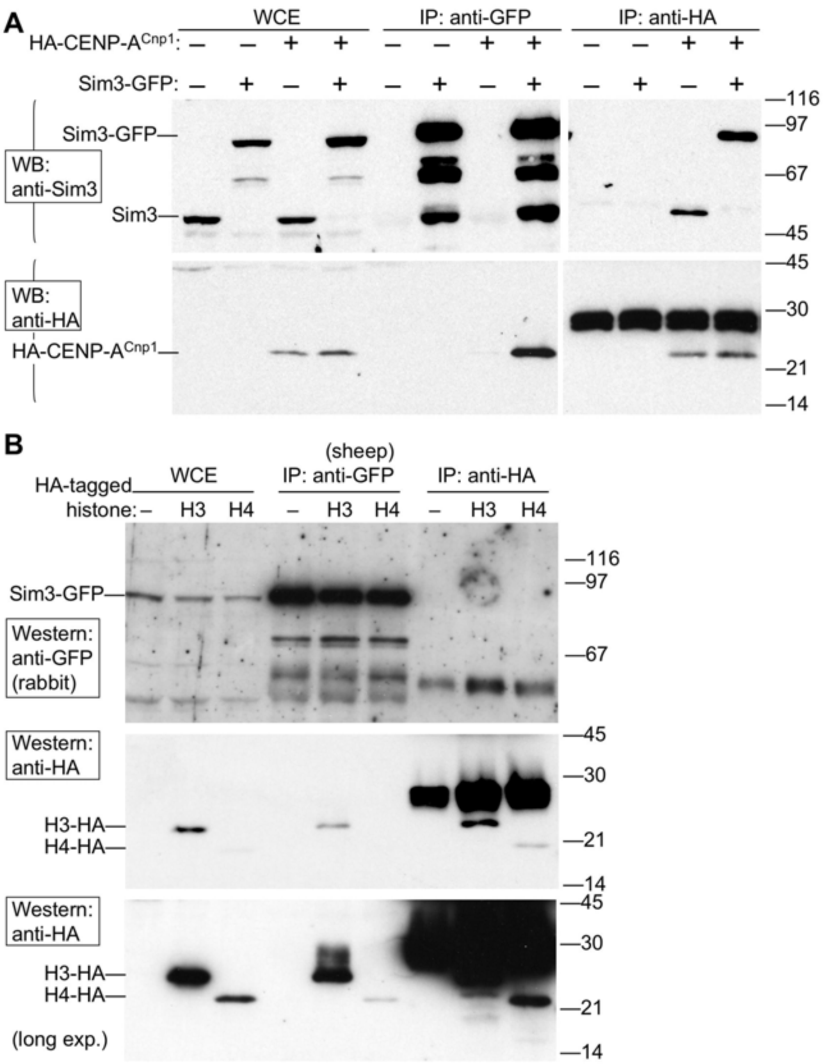

Figure S6

A. Overexpressed HA-CENP-A^{Cnp1} (from *nmt41x* promoter) co-immunoprecipitates with Sim3-GFP (IP: sheep anti-GFP). Reciprocally, Sim3-GFP and untagged Sim3 co-immunoprecipitate with overexpressed HA-tagged CENP-A^{Cnp1} (IP: mouse anti-HA). Strains used: FY1645, FY6322, FY4115, FY11058.

B. Overexpressed histones H3-HA and H4-HA (from p*inv1*-H3-HA, or p*inv1*-H4-HA; Choi et al, 2005) co-immunoprecipitates with Sim3-GFP. Sim3-GFP is not detected in reciprocal anti-HA (H3/H4) immunoprecipitates. Strains used: FY6322 containing indicated plasmids.

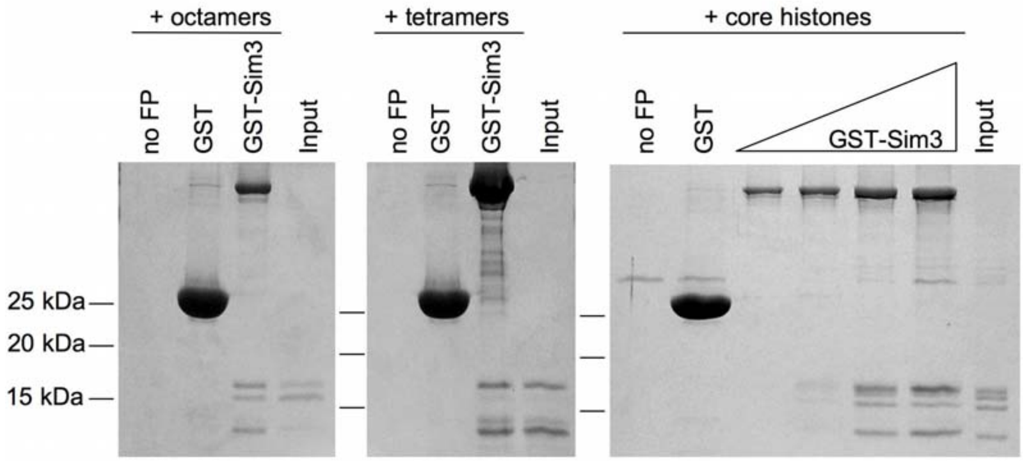

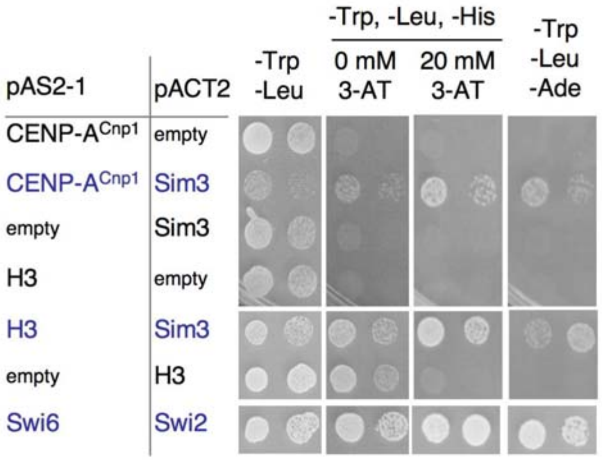

Figure S7

Top: GST-Sim3 or GST alone was incubated with recombinant histone H3-H4-H2A-H2B octamers, H3-H4 tetramers or free core histones from Calf Thymus (Upstate). GST-Sim3 has an affinity for all four histone regardless of the source. BSA was added as a competitor in binding assays with free core histone (right panel). Upper histone band is H3, then H2B, then H2A and lowest is H4. GST-Sim3 may enrich for H3 over H2A and H2B in binding assays with free histones (right panel).
Bottom: Two Hybrid assay in *S. cerevisiae* with strains expressing CENP-A^{Cnp1}, H3 or Swi6 fused to the Gal4 DNA Binding domain (GBD) or GDB alone in PAS2-1 and therefore targeted to the Gal4 sites residing adjacent *HIS2* and *ADE2* genes in the tester strain. Strains also express Sim3, H3, or Swi2 fused to the Gal4 activation domain (ACT) or ACT alone in pACT2. Interactions are indicated by increased growth on plates lacking Histidine (-His) and containing 20 mM 3-AT or on plates lacking adenine (-Ade). Two hybrid plasmids were selected on plates lacking tryptophan and leucine (-Trp –Leu). The Swi6-Swi2 interaction serves as a positive control.

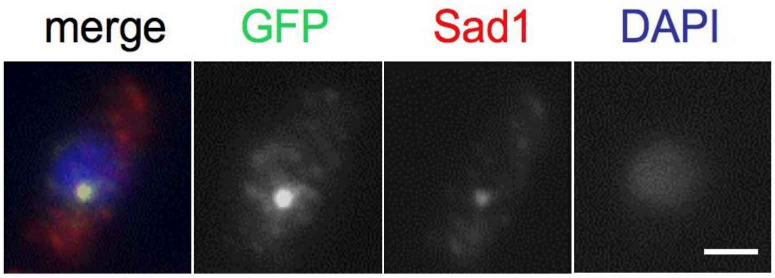

Figure S8

Newly induced GFP-CENP-A^{Cnp1} spots are localised at centromeres. *inv*-GFP-CENP-A^{Cnp1} was induced in wild type cells for 1 hour at 25°C and cells were fixed and co-stained with anti-GFP (green), anti-Sad1 (red) to mark the SPB and DNA was stained with DAPI (blue). Strain FY8481.

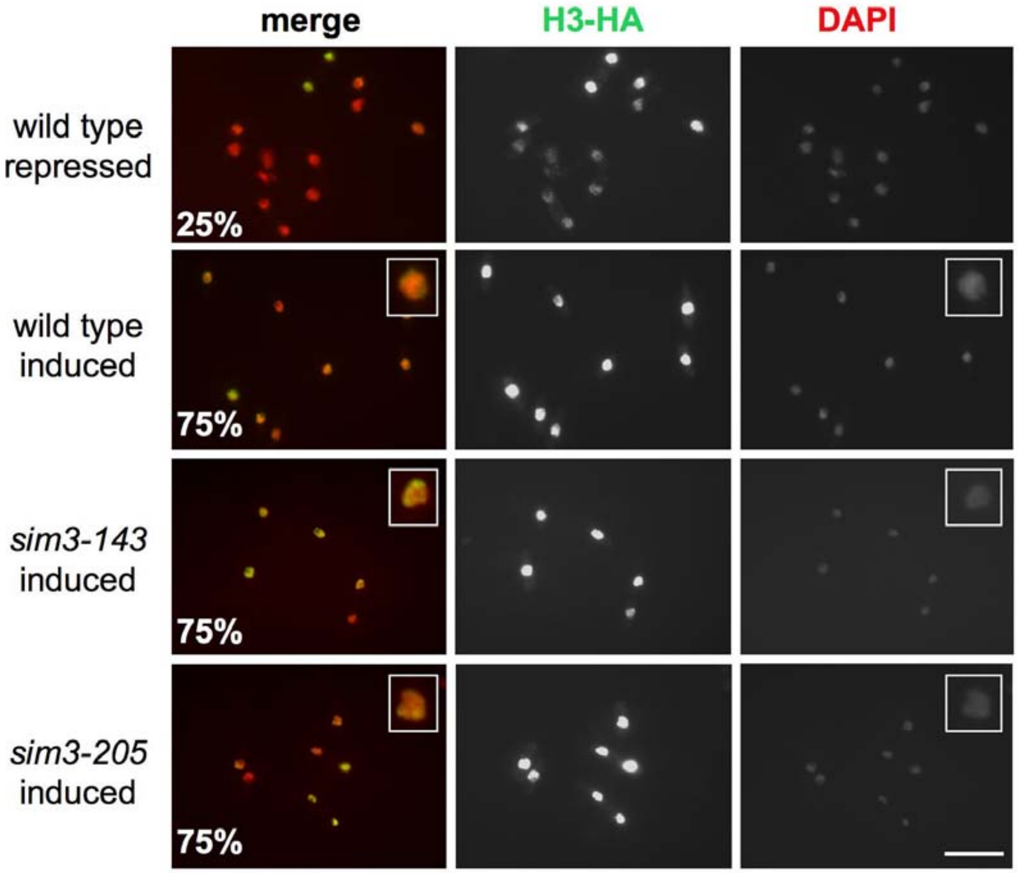

Figure S9

Localisation of newly induced HA tagged histone H3 in wild type and *sim3* mutants at 25°C.
Wild type and sim3 mutants containing the p*inv*-H3HA plasmid (Choi et al., 2005) were shifted from repressed to inducing conditions 1 hour prior to fixation and immunolocalisation with anti-HA antibody (green) and staining of DNA (red). The percentage of cells in which H3-HA localised to chromosomal DNA was determined (n=200). Scale bar, 10 μm.

Table S1
Microarray expression profiling analysis of *sim3* mutants

**HIGH expression in *sim3-205* versus WT. 2-fold cut off (3 of 4 data points)** 16 genes	
**Gene Name**	**Gene dB Product description**
SPCC23B6.02c	hypothetical lysine-rich protein; sequence orphan
SPCC330.03c	putative hypothetical heme binding protein
SPCC338.12	putative proteinase precursor
SPCC757.13	putative MFS allantoate transporter
tf2-10	
SPBC3F6.06c	
SPBC1677.02	putative dolichol phosphate mannose synthase; can weakly stabilize mammalian DPM1 protein; no S. cerevisiae homolog
SPBC16A3.07c	hypothetical protein.; sequence orphan
SPBC16C6.09	putative dolichyl-phosphate-mannose--protein mannosyltransferase
SPBC1683.06c	putative inosine-uridine preferring nucleoside hydrolase
SPBC1685.05	putative serine protease
SPBC16D10.08c	putative chaperonin; heat shock protein
SPBC16E9.16c	pseudogene
SPBC3F6.04c	conserved hypothetical protein; similar to S. cerevisiae YDL148C which purifies as part of the nuclear pore complex and localises to the nucleus and portion of the nucleolus
SPBC4C3.04c	putative guanine nucleotide exchange factor; possible yeast dss4 homolog- Guanine-nucleotide exchange factor for Sec4p
SPBC543.10	putative coiled-coil protein similar to human congenital heart disease 5 protein chd5; similar to S. cerevisiae YGL020C

**LOW expression in *sim3-205* versus WT. 2-fold cut off (3 of 4 data points)** 4 genes	
**Gene Name**	*Gene dB Product description*
prp28	putative pre-mRNA splicing factor; U5 snrnp-like RNA helicase subunit; similar to S. cerevisiae PRP28
rpp0	putative 60s acidic ribosomal protein p0
SPAC2E1P3.05c	putative cellulose binding beta glucosidase
meu14	RING zinc finger; putative coiled-coil region; meiotic expression up regulated

**HIGH expression in *sim3-143* versus wt. 1,5 fold cut-off (3 of 4 data points)** 23 genes	
**Gene Name**	**Gene dB Product description**
thi3	pyrimidine precursor biosynthesis enzyme thi3

SPCC191.05c	hypothetical protein; sequence orphan; has transcript profile on microarray
SPCC320.02c	
SPCC663.06c	putative short chain dehydrogenase protein
SPCC794.15	very hypothetical protein
SPCC965.11c	APC amino acid transporter
apl5	putative Delta-adaptin, large subunit of the clathrin-associated protein (AP) complex
SPAC1F8.04c	putative chlorohydrolase/deaminase; putative guanine deaminase
SPAC212.11	DNA helicase possibly pseudo
SPAC631.02	bromodomain protein; similar to S. cerevisiae BDF1
SPAC13G7.02c	heat shock protein 70
SPAC23C4.07	hypothetical protein; sequence orphan
vps17	putative vacuolar sorting protein
orp4	origin recognition complex subunit 4
SPBC28F2.05c	putative oxidoreductase
SPBC36B7.04	protein with weak similarity to Azospirillum brasilense nifR3 protein; yeast YML080W homolog; UPF0034 domain
SPBC36B7.06c	hypothetical protein; sequence orphan
SPBC3E7.11c	DNA J domain protein
SPBC8E4.02c	hypothetical protein; sequence orphan
SPBP4G3.03	hypothetical protein; possibly S. pombe specific; similar at the N-term to SPBP7G5.01
SPBP8B7.29	probable para-aminobenzoate synthase
tms1	putative sorbitol dehydrogenase
tor2	

**LOW expression in *sim3-143* versus wt. 1,5 fold cut off (3 of 4 data points)** 18 genes	
**Gene Name**	**Gene db Product description**
adh1	alcohol dehydrogenase I; reducing acetaldehyde to ethanol, regenerating NAD+
mms2	ubiquitin conjugating enzyme; ubc
par1	Protein phosphatase PP2A, subunit B', required for normal cytokinesis, morphogenesis, and stress tolerance
SPCC1322.10	hypothetical protein; sequence orphan; serine-rich protein; similar to cell-surface proteins; similar to proteoglycans
eng1	endo-1,3-beta-glucanase
SPAC1A6.04c	putative lysophospholipase precursor
nuc1	dna-directed rna polymerase i 190 kd polypeptide
SPAC17H9.14c	putative protein disulfide isomerase (EC 5.3.4.1)
SPAC19G12.08	putative fatty acid hydroxylase
SPAC1D4.08	cdp-diacylglycerol--inositol 3-phosphatidyltransferase
SPAC222.08c	Putative pyridoxine (vitamin B6) biosynthetic enzyme; similar to glutamine aminotransferases by similarity to yeast sno1,2 and 3
SPAC23H3.09c	putative threonine aldolase
SPAC630.08c	putative c-4 methyl sterol oxidase
SPBC28E12.03	GTPase-activator protein for Rho-like GTPases
SPBC1347.09	putative methyltransferase; similar to S. cerevisiae coq3

SPBC16A3.10	hypothetical protein; similar to S. cerevisiae YOR175C
SPBC16E9.07	hypothetical protein; sequence orphan
SPBC17G9.06c	putative acetyltransferase;Pfam-B_12207; Pfam-B_5757; similar to bacterial siderophore biosynthesis protein

**HIGH expression in *sim3-205* versus wt 1,5 fold (3 of 4)** 68 genes	
**Gene Name**	*Gene dB Product description*
SPCC417.05c	putative involvement in chitin biosynthesis; by similarity
SPCC191.09c	putative glutathione s-transferase
SPCC965.07c	Glutathione S-transferase
SPCC188.11	putative chromatin binding snw family nuclear protein; putative spliceosome associated by similarity to human skip/snw1
rpl39	60s ribosomal protein l39
SPCC18B5.04	mitochondrial ribosomal protein S18
SPCC191.04c	very hypothetical protein
SPCC1919.03c	putative 5'-amp-activated protein kinase; putative regulation of fatty acid synthesis by the phosphorylation of acetyl-Coa carboxylase; possibly also regulates carbohydrate metabolism by similarity to S. cerevisiae SIP2- a protein that interacts with SNF1
SPCC23B6.02c	hypothetical lysine-rich protein; sequence orphan
SPCC24B10.05	similar to S. cerevisiae Tim9p essential component of the mitochondrial import machinery; required for transfer of hydrophobic inner membrane carrier proteins across the intermembrane space
SPCC24B10.16c	hypothetical protein; sequence orphan; has transcript on microarray
SPCC330.03c	putative hypothetical heme binding protein
SPCC338.12	putative proteinase precursor
SPCC417.09c	putative zinc-finger protein
SPCC417.13	
SPCC70.08c	probable methyltransferase
SPCC70.09c	hypothetical protein; similar to S. cerevisiae YMR295C and YGR273C (low) ;similar to S. pombe SPBC19C7.04c; possibly fungal specific
SPCC965.06	putative potassium channel subunit
SPCC965.14c	putative Cytidine and deoxycytidylate deaminase zinc-binding protein
SPCC576.01c	hypothetical protein; sequence orphan; very low similarity to alpha-ketoglutarate-dependent taurine dioxygenase
SPCC24B10.14c	hypothetical protein; sequence orphan
rpc19	Putative shared subunit of RNA polymerases I and III, has similarity to S. cerevisiae Rpc19p
SPAC1039.08	serine acetyltransferase
SPAC17G8.11c	protein with similarity to S. cerevisiae SUR1 which is required for the synthesis of mannosylated sphingolipids
SPAC186.07c	2-hydroxyacid dehydrogenase
SPAC186.08c	putative l-lactate dehydrogenase

rdp1	zf-C2H2, Zinc finger; regulator of rhp51; promoter has damage responsive elements (DREs)
SPAC1B3.20	hypothetical protein with confirmed intron; sequence orphan
SPAC1D4.09c	conserved hypothetical protein; no S. cerevisiae homologue
SPAC1F12.10	putative heme binding protein; putative flavoprotein
SPAC1F8.04c	putative chlorohydrolase/deaminase; putative guanine deaminase
SPAC227.05	putative gim complex; prefoldin subunit 4
SPAC23H3.11c	putative beta-glucan synthesis-associated protein
tf2-10	
SPBC56F2.10c	dolichyl-phosphate beta-glucosyltransferase
SPBC3F6.06c	
SPBC1604.11	putative F-type ATPase subunit F; mitochondrial
SPBC1677.02	putative dolichol phosphate mannose synthase; can weakly stabilize mammalian DPM1 protein; no S. cerevisiae homolog
SPBP8B7.12c	hypothetical protein; sequence orphan
SPBC16E9.13	putative serine/threonine protein kinase; similar to S. cerevisiae KSP1
SPBC16A3.07c	hypothetical protein.; sequence orphan
SPBC3B9.16c	putative nucleoporin
SPBC16C6.09	putative dolichyl-phosphate-mannose--protein mannosyltransferase
SPBC577.05c	hypothetical protein; sequence orphan
rps27	40s ribosomal protein s27
smg1	small nuclear ribonucleoprotein g; putative pre-mRNA splicing factor; similar to S cerevisiae SMX2
SPBC146.06c	conserved hypothetical protein; similar to O sativa Q9LWI8; Arabidopsis thaliana F11A17.9; human KIAA1018 and C elegans C01G5.8; has conserved motifs at C terminal one : DY{K/R}{S/G}.PDL..W{.*}EVK...D{R/Q}LS{EHT}.Q..W{I?L}..{L/F}.VE{I/V}C{K/HV
SPBC14F5.02	hypothetical protein; sequence orphan
SPBC1683.05	NCS1 allantoate transporter
SPBC1683.06c	putative inosine-uridine preferring nucleoside hydrolase
SPBC1683.12	MFS transporter of unknown specificity
SPBC1685.05	putative serine protease
SPBC16A3.12c	triglyceride lipase-cholesterol esterase.
SPBC16A3.19	hypothetical protein; similar to drosophilla CG13746;subcellular localization of GFP fusion-nucleus
SPBC16D10.08c	putative chaperonin; heat shock protein
SPBC16E9.16c	pseudogene
SPBC16G5.04	mitochondrial ribosomal protein L23
SPBC16G5.16	fungal Zn(2)-Cys(6) binuclear cluster zinc finger transcription factor
SPBC216.04c	putative transcriptional regulator
SPBC3E7.07c	hypothetical protein; similar to S. cerevisiae YPL225W
SPBC3F6.04c	conserved hypothetical protein; similar to S. cerevisiae YDL148C which purifies as part of the nuclear pore complex and localises to the nucleus and portion of the nucleolus

SPBC4.01	hypothetical protein; sequence orphan; contains 4 predicted transmembrane helices contains predicted N-term signal peptide
SPBC418.03c	
SPBC4C3.04c	putative guanine nucleotide exchange factor; possible yeast dss4 homolog- Guanine-nucleotide exchange factor for Sec4p
SPBC543.10	putative coiled-coil protein similar to human congenital heart disease 5 protein chd5; similar to S. cerevisiae YGL020C
SPBC887.01	hypothetical protein; similar to S. cerevisiae YMR009W
SPBPB10D8.04c	putative malate permease
SPBC4F6.09	MFS efflux transporter of unknown specificity

**LOW expression in *sim3-205* versus wt. 1,5 fold (3 of 4 data points)** 45 genes	
**Gene Name**	**Gene dB Product description**
prp28	putative pre-mRNA splicing factor; U5 snrnp-like RNA helicase subunit; similar to S. cerevisiae PRP28
rpp0	putative 60s acidic ribosomal protein p0
SPCC1442.12	cdp-diacylglycerol--serine o-phosphatidyltransferase
ade3	phosphoribosylformylglycinamidine synthase; subcellular localization of GFP fusion- Cytoplasm
SPAC14C4.09	putative Glucanase; by similarity to Penicillium purpurogenum mutanase; subcellular localization of GFP fusion- Membrane
cka1	casein kinase ii, alpha chain; involved in cell growth regulation
clp1	putative dual specificity protein-tyrosine phosphatase (PTPase); possibly S phase; involved in septation; similar to S. cerevisiae CDC14
dak1	dihydroxyacetone kinase; isoenzyme I; subcellular localization of GFP fusion; Cytoplasmic dots and lines
n313	aminomethyltransferase precursor; Glycine decarboxylase T subunit
orp3	origin recognition complex subunit 3
pab1	poly(A) binding protein; polyadenylation factor
psu1	cell wall synthesis protein psu1
rad15	
rps10-1	40s ribosomal protein s10
SPAC12B10.05	putative metallopeptidase
SPAC12B10.09	MC transporter of unknown specificity
SPAC14C4.12c	hypothetical protein; similar to S. cerevisiae YAL034C
SPAC16.05c	hypothetical zinc finger protein
SPAC16E8.01	putative cytoskeleton assembly control protein; SRC homology domain
SPAC1782.06c	putative prohibitin complex subunit; possibly involved in protein degradation and determination of replicative lifespan; similar to S. cerevisiae PHB1
SPAC17A2.10c	very hypothetical protein
SPAC2C4.10c	hypothetical protein; sequence orphan
SPAC2E1P3.05c	putative cellulose binding beta glucosidase

SPAC3A11.07	putative nadh-dehydrogenase
SPAC3A11.09	CPA1 sodium ion/proton antiporter
SPAC3H5.08c	WD repeat protein
SPAC4G8.07c	putative Uridine methyltransferase that catalyzes the formation of ribothymidine in tRNAs; by similarity to trm2
SPACUNK4.09	hypothetical product; sequence orphan
SPAPB1A10.13	hypothetical protein; similarity to proteoglycans
exg3	glucan 1,3-beta-glucosidase
meu14	meu14. RING zinc finger; putative coiled-coil region; meiotic expression upregulated
pgr1	glutathione reductase
php2	CCAAT-box binding factor subunit; required for growth on non-fermentable carbon sources; transcriptional activator; similar to S. cerevisiae HAP2
prp17	WD repeat protein; putative pre-mRNA splicing factor; similar to S. cerevisiae CDC40
SPBC119.10	asparagine synthetase
SPBC1198.08	hypothetical protein; similar to S. cerevisiae YFR044C
SPBC18E5.12c	putative mitochondrial processing peptidase alpha subunit
SPBC21D10.10	bromodomain protein
SPBC30D10.01	zuotin like protein; putative zdna binding; dnaj domain containing protein
SPBC342.02	glutaminyl-trna synthetase, mitochondrial
SPBC359.03c	Putative amino acid permease
SPBC409.08	putative membrane transport protein
SPBC6B1.05c	putative protein involved in autophagy; S. cerevisiae APG7 homolog is involved in vacuole targeting and peroxisome degradation
SPBP35G2.11c	protein containing 3 ZZ domain zinc finger domains
psu1	cell wall synthesis protein psu1
SPBC1706.01	SH3 Src homology domain protein

Supplementary Experimental Procedures

Media and yeast culture

Chemicals were obtained from Sigma-Aldrich (St. Louis, MI) unless stated otherwise. Culture of fission yeast followed standard protocols (Moreno et al., 1991).

Yeast strain construction

A list of *S. pombe* strains used in this study is shown in Table S2. Strain FY8481 was constructed as follows: the *inv1* promoter was isolated by PCR as a 2.3 kb *BamHI-PstI* fragment (313 and 314 primer pair), GFP-CENP-A^{Cnp1} was amplified from genomic DNA from strain FY3917 (B. Mellone) containing integrated pREP42-EGFP-*CENP-*A^{cnp1+} (315 and 318 primer pair) and was cloned downstream of the *inv1* promoter into a 'split URA' plasmid in which the *ura4* gene is disrupted by the insertion of restriction sites. A linearised fragment containing P*inv1*-GFP-CENP-A^{Cnp1} with *ura4* sequences at the 5' and 3' ends was then integrated into a wild type strain (FY96) at *ura4*$^{+}$ locus. FOA^{R} colonies were checked for correct integration of P*inv1*-GFP-CENP-A^{Cnp1} at *ura4* locus (WA03 and pINV-seq1 primer pair). The endogenous CENP-A^{Cnp1} ORF was tagged at its N terminus with 5 copies of the myc epitope tag using a two step strategy which replaced *cnp1::ura4*$^{+}$ with the tagged ORF. *sim3*$^{+}$ was C-terminally tagged in the genome with GFP by cloning a 650 bp *Kpn*I-*Sal*I fragment from the 3' end of the sim3 ORF (obtained by PCR with primers 167 and 168) into plasmid pDM84 (Millband and Hardwick, 2002) which contains GFP and *his3*$^{+}$. The resulting plasmid was linearised with *Pst*I which cuts in the *sim3* region and transformed into wild-type and *sim3-143* mutant strains, with selection for growth on –histidine plates. Correct integration was confirmed by PCR. Sim3 tagged with GFP was functional since strains containing it as the only source of Sim3 grew as wild-type at all temperatures and had intact central core silencing. The *sim3-143-GFP* strain displayed the same phenotypes as an untagged *sim3-143* strain in these assays. CENP-A^{Cnp1} was tagged with GFP by inserting the *cnp1*$^{+}$ ORF into pREP42X-N-GFP. The resulting plasmid was linearised at the *ars1* element by cutting with *Mlu*I, and used to transform *S. pombe*; correct integration at *ars1* was confirmed by PCR. These strain (5205/5206) also contain endogenous *cnp1*$^{+}$. Strain 4115 expressing HA-tagged CENP-A^{Cnp1} from integrated pREP41X-*LEU2* was constructed in an analogous manner.

A diploid with the *sim3*$^{+}$ ORF replaced with the KanMX cassette (obtained from Bioneer) was spoulated and tetrads dissected. Slow growing *sim3Δ*::KanMX (*sim3Δ*) haploids were isolated and further characterised.

Plasmids and primers

A list of the primers used in this study is shown in Table S2. For recombinant protein expression in bacteria, Sim3, CENP-A^{Cnp1}, H3, H4, H2A and H2B ORFs were cloned into pGEX-4T1. For pREP41X-CENP-A^{Cnp1}, -H3 and -H4, ORFs were amplified by PCR and fragments were cloned as *Xho*I/*Bam*HI fragments. Constructs were verified by sequencing

Production of anti-Sim3 antibody

sim3^{+} cDNA was PCR amplified (198 and 199 primer pair) and cloned into pGEX-4T1 (Amersham Biosciences). GST-Sim3 fusion protein was purified and was used to immunize a rabbit. Anti-Sim3 antibodies were affinity purified against cleaved Sim3 (using Thrombin CleanCleave kit; Sigma) on nitrocellulose.

Western blotting

Antibodies for Western blotting were diluted in PBS + 0.2% Tween as follows: anti-Sim3, 1:300 to 1:1000; rabbit anti-Myc (Research Diagnostics) 1:1000; rabbit anti-myc 1:1000 (A14; Santa Cruz); rabbit anti-GFP (Molecular Probes) or rabbit anti-GFP (gift from K. Hardwick) 1:1000 (in PBS-Tween, 1% dried milk); mouse anti-GFP (BD Living Colours) 1:500; sheep anti-GFP (gift from K. Hardwick) 1:2000; mouse TAT1 anti-tubulin (gift from I.Hagan) 1:500; rabbit anti-H3C (1791; AbCam) 1:2,500; rabbit anti-Bip1 (A. Pidoux) 1:5000; mouse anti-HA (12CA5; gift from Kumiko Samejima). Blots were developed using ECL reagents (Amersham Biosciences).

Immunoprecipitations

Immunoprecipitations were performed as described (Millband and Hardwick, 2002), with modifications. Cell pellets from log-phase cultures were resuspended in lysis buffer (50 mM HEPES pH 7.5, 150 mM NaCl, 1 mM $MgCl_2$ 1 mM EGTA, 0.1% NP-40, 2 mM PMSF, protease inhibitor cocktail for yeast (Sigma)) at 1 x 10^9 cells/ml and ground in liquid nitrogen with a pestle and mortar. Lysates were spun to remove

debris and precleared. IPs were performed on extract from 5 x 10^8 cells at 4°C for 3-18 hours, using 20 μl anti-myc(9E10)-agarose (Santa Cruz), 10 μl sheep anti-Cnp1 serum, 2 μl affinity purified sheep anti-GFP antibody (a gift from Kevin Hardwick), 10 μl affinity purified rabbit anti-Sim3 antibody, and Protein A or G agarose (Roche) as appropriate. Immunoprecipitates (IPs) were washed three times with ice-cold lysis buffer and twice with PBS. SDS-PAGE and Western blotting was used for analysis of IPs. For Figures 5A and S6, whole cell extract (WCE) from 2 x 10^6 cells or immunoprecipitated material from ~1 x 10^8 cells was loaded per lane. Exposure times for ECL-developed westerns varied. For Figure 5C, extracts or immunoprecipitates from the following numbers of cells were loaded. Anti-myc western: WCE, 2 x 10^6; sheep anti-GFP IP, 1 x 10^8; mouse anti-myc (9E10) IP, 1 x 10^8. Anti-Sim3 western: WCE, 4 x 10^5; sheep anti-GFP IP, 2 x 10^6; mouse anti-myc (9E10) IP, 1 x 10^8.

***In vitro* binding assays**

^{35}S-labelled Sim3 was produced using primers 202 and 199 and a TNT T7 Quick for PCR DNA kit (Promega) according to manufacturer's instructions. For *in vitro* binding, 4 μg GST fusion protein and 10 μl of ^{35}S-labelled Sim3 were incubated on ice for 30 minutes in binding buffer (50 mM HEPES pH 7.6, 75 mM KCl, 1 mM $MgCl_2$, 1 mM EGTA, 0.5 mM DTT, 1 mM PMSF, Protease inhibitors), incubation for 1 h with glutathione-agarose followed by 4 washes in binding buffer. Samples were analysed by SDS-PAGE and fluorography. 2 μl (i.e. 1/5th) of ^{35}S-labelled Sim3 was loaded in the non-pull-down lanes.

Chromatin Immunoprecipitation (ChIP)

10 μl anti-CENP-A^{Cnp1} antiserum, 2-4 μl rabbit anti-H3C (AbCam, ab1791) and 1.5 μl rabbit anti-GFP (Molecular Probes) were used per ChIP (2.5 x 108 cells; 400 μl extract). Multiplex PCR analysis was performed as described previously (Pidoux et al., 2003). Quantitation of PCR product bands was performed using the Kodak EDAS 290 system and 1D Image Analysis Software (Eastman Kodak). For the input PCR the *cnt1* and *imr1* values were normalised to the *fbp1* value, giving the 'input ratio'. Enrichment of *cnt1* (TMA and TMB primer pair), *imr1* (IMRA and IMRB primer pair) bands in the ChIPs was calculated relative to the *fbp1* (FBPA and FBPB primer pair) band, and then corrected for the ratio obtained in the input PCR. ChIP experiments were performed 2-5 times; representative examples are presented.

Quantitative Real Time PCR on a Biorad iCycler was used to assess the relative association of CENP-A^{Cnp1} with *cnt1* relative to *act1* in wild-type, *sim3-143* and *sim3-205* samples at 25°C and 36°C. Reactions were performed with SYBR Green Jumpstart Taq ReadyMix (Sigma) with primers specific for *cnt1* or *act1*.

Cytology

The following antibodies were used: sheep anti-CENP-A^{Cnp1} antiserum 1:300 to 1:2000; rabbit anti-Sad1 1:40 (provided by Iain Hagan); mouse TAT1 anti-tubulin 1:15 mouse (Iain Hagan, Keith Gull); 12CA5 anti-HA 1:30; affinity purified anti-Sim3 1:40; rabbit anti-GFP (Molecular Probes) 1:50. Alexa Fluor 594 (Invitrogen, A11016) or Alexa Fluor 488 (Invitrogen, A11029 or A21441)-conjugated secondary antibodies were used at 1:1000. Cells expressing GFP-CENP-A^{Cnp1} were fixed in freshly prepared 3.7% formaldehyde for either 20 minutes at 25°C or 5 minutes at 36°C and were analysed by fluorescent microscopy. Microscopy was performed as described in Pidoux et al., 2003. Image acquisition was controlled using Metamorph software (Universal Imaging Corporation).

Northern Blotting

For Northern blotting, 800 bp GFP and 1 kb *adh1* probes were amplified from genomic DNA, were synthesised using High Prime (Roche) and were hybridised overnight at 55°C.

Structural alignments

The following sequences were aligned: S.p. (Sim3 *S. pombe* NP_595313), S.j. (*Schizosaccharomyces japonicus* [DNA] 220518-221633), C.a. (Candida albicans XP_71223), N.c. (*Neurospora crassa* XP_961229), A.t. (*Arabidopsis thaliana* NP_568019), C.e.(*C. elegans* NP_496380), D.m. (*D. melanogaster* NP_649828), X.l (*Xenopus laevis*, AAH7744), H.s. (Homo sapiens NASP NP_689511), S.c (*S.cerevesiae* Hif1 Q12373). The four TPR motif shown in Figure 2C was made by ratcheting the first motif along to superpose on the second such that the new position of the third is used as a model for the fourth.

Expression Profiling

cDNA expression profiling was carried out according to (Xue et al., 2004). We used the *S. pombe* ORF spotted microarrays containing 5029 ORF probes (Eurogentec, Belgium). Gene expression in logarithmically growing cultures of *sim3* mutant cells was compared to that in wt controls to establish lists of genes affected by *sim3-143* and *sim3-205*. 'GeneSpring' software (Agilent) was used for all the data analysis. The 'Lowess' (per spot per chip) intensity-dependent normalization, which corrects nonlinear rates for dye incorporation, was used. The 'Gene dB' product descriptions for the affected genes were downloaded from www.genedb.org/genedb/pombe/ and linked to the gene names using Microsoft Access (See Table S1). The Gene Expression omnibus (GEO) submission series for the *sim3* microarray data is GSE7560 at http://www.ncbi.nlm.nih.gov/geo/.

Table S2: Strains used in this study

For strains marked with an asterisk (*) only the relevant genotype is listed.

Strain	Genotype
972	*h^{-}*
96	*h^{-} leu1-32 his3- ade6-216*
1645	*h^{+} ade6-210 arg3-D4 his3-D1 leu1-32 ura4-D18*
1646	*h^{-} ade6-210 arg3-D4 his3-D1 leu1-32 ura4-D18*
3027	*h^{+} cnt1:arg3^{+} otr2:ura4^{+} cnt3:ade6^{+} tel1:his3^{+} ade6-210 arg3-D4 his3-D1 leu1-32 ura4-D18/DS-E*
3033	*h^{-} cnt1:arg3^{+} otr2:ura4^{+} cnt3:ade6^{+} tel1:his3^{+} ade6-210 arg3-D4 his3-D1 leu1-32 ura4-D18/DS-E*
3606	*h^{-} rik1::LEU2^{+} cnt1:arg3^{+} otr2:ura4^{+} cnt3:ade6^{+} tel1:his3^{+} ade6-210 arg3-D3 his3-D1 leu1-32 ura4-D18/DS-E*
4115	*h^{+} cnp1Δ::ura4^{+} ars1(MluI):pREP41X-HA-cnp1-LEU2 ade6-216 leu1-32 ura4-D18*
4462	*h^{-} cnp1-76 cnt1:arg3^{+} otr2:ura4^{+} cnt3:ade6^{+} tel1:his3^{+} ade6-210 arg3-D4 his3-D1 leu1-32 ura4-D18/DS-E*
5205/6	*h^{+} ars1(MluI):pREP42X-GFP-cnp1-ura4+ leu1-32* (*)
5496	*h^{-} sim3-205 cnt1:arg3^{+} otr2:ura4^{+} cnt3:ade6^{+} tel1:his3^{+} ade6-210 arg3-D4 his3-D1 leu1-32 ura4-D18/DS-E*
5691	*h mis6-302 cnt1:arg3^{+} otr2:ura4^{+} cnt3:ade6^{+} tel1:his3^{+} ade6-210 arg3-D4 his3-D1 leu1-32 ura4-D18/DS-E*
5927	*h^{+} 5myc-cnp1 leu1-32 ura4-D18 his3-D1 ade6-210*
6154	*h^{+} sim3-143 cnt1:arg3^{+} otr2:ura4^{+} cnt3:ade6^{+} tel1:his3^{+} ade6-210 arg3-D3 his3-D1 leu1-32 ura4-D18/DS-E*
6308	*h^{-} sim3-143-GFP-his3^{+} cnt1:arg3^{+} otr2:ura4^{+} cnt3:ade6^{+} tel1:his3^{+} ade6-210 arg3-D3 his3-D1 leu1-32 ura4-D18/DS-E*
6322	*h^{-} sim3-GFP-his3^{+}* ade6-210 ura4D18 leu1-32 his3D1 arg3D4
6326	*h^{+} sim3^{+}-GFP-his3^{+} cnt1:arg3^{+} ade6-210 ura4-D18 leu1-32 his3-D1 arg3-D4*
6368/9	*h sim3-143-GFP-his3^{+} 5myc-cnp1 (*)*
6374/5	*h sim3-GFP-his3^{+} 5myc-cnp1 (*)*
6443	*h^{+} hht2-GFP-ura4^{+} leu1-32 ade6-210*
7460	*h sim3-143 5myc-cnp1 leu1-32 ura4-D18 his3-D1 ade6-210* (*)
7461	*h sim3-205 5myc-cnp1 leu1-32 ura4-D18 his3-D1 ade6-210* (*)

8481 *h⁻ ura4::inv-GFP-cnp1 leu1-32 his3-D1 ade6-216* (*)

8482 *h sim3-143 ura4::inv-GFP-cnp1 leu1-32* $his3^-$ (*)

8483 *h sim3-205 ura4::inv-GFP-cnp1 leu1-32* $his3^-$ (*)

8519 *h mis6-302 ura4::inv-GFP-cnp1 leu1-32* $his3^-$ (*)

8517 *h cdc10-129 ura4::inv-GFP-cnp1 leu1-32* (*)

8518 *h cdc25-22 ura4::inv-GFP-cnp1 leu1-32* (*)

8717 *h cdc25-22 sim3-143 ura4::inv-GFP-cnp1 leu1-32 his3-* (*)

8718 *h cdc25-22 sim3-205 ura4::inv-GFP-cnp1 leu1-32 his3-* (*)

6960 h^- *cnp1Δ::*$ura4^+$ $lys1^+$*::cnp1-1 leu1-32*

11058 *h sim3-GFP-*$his3^+$ *cnp1Δ::*$ura4^+$ *ars1(Mlu***I***):pREP41X-HA-cnp1-LEU2* (*)

11049 *h sim3Δ::kanMX6* $ade6^-$ *ura4-D18 leu1-32*

11052 *h* $sim3^+$ $ade6^-$ *ura4-D18 leu1-32*

10539 h^+ $lys1^+$*(cen1):lacO D107(cen2):kanMX6-ura4+-lacO* $his7^+$*:lacI-GFP(*)*

11055 *h sim3-205* $lys1^+$*(cen1):lacO D107(cen2):kanMX6-ura4+-lacO* $his7^+$*:lacI-GFP (*)*

Table S3: Primers used in this study

198	sim3-5-EcoR1	TACTACGAATTCATGTCTTCTGATACGAAAACACTG
199	sim3-3-Xho1	TACTACCTCGAGTTAATCCTTCTTTTTCTTATCTTTAGGACC
202	sim3-5-T7kz	GTTGTGTTTAATACGACTCACTATAGGGCGAGAGCCACCATGTC ATGTCTTCTGATACGAAAACACTG
26	TM1A	AACAATAAACACGAATGCCTC
27	TM1B	ATAGTACCATGCGATTGTCTG
33	FBPA	AATGACAATTCCCCACTAGCC
34	FBPB	ACTTCAGCTAGGATTCACCTGG
	IMRA	GGCTACCAGCATTGTTATTCATAA
	IMRB	GGATATATGTATTCTTGCACTC
313	pINV-5-BamHI	AAAAACTGCGGATCCACTTTTGATCCGTTC
314	pINV-3-PstI	TACTACCTGCAGGCAAATCTTCAAAGTTAG
315	GFP-5-PstI	TACTACCTGCAGAGTAAAGGAGAAGAACTT
318	cnp1-3-EcoR1	TACTACGAATTCTCAAGCACCACGAATCCT
	WA03	CCCACTGGCTATATGTATGCATTT
	PINV-seq1	CCGCCATTGCACTCAGCCATAC
	GFP_for	GTAAAGGAGAAGAACTTTTCACTG
	GFP_rev	TCATCCATGCCATGTGTAATC
200	cnp1-5-NdeI-RI	TACTACCATATGGAATTCATGG CAAAGAAATCTTTAATGGC
201	cnp1-3-XhoBm	TACTACCTCGAGGGATCCTCAA GCACCACGAATCCTCCTGG
168	sim3-5-K-84	TACTACGGTACCGTCATGCAGTCAAAAGCAGTAGATGC
169	sim3-3-XS-84	TACTACCTCGAGGTCGACATCCTTCTTTTTCTTATCTTTAGGACC

Supplementary references

Choi, E.S., Shin, J.A., Kim, H.S., and Jang, Y.K. (2005). Dynamic regulation of replication independent deposition of histone H3 in fission yeast. Nucleic acids research 33, 7102-7110.

Millband, D.N., and Hardwick, K.G. (2002). Fission yeast Mad3p is required for Mad2p to inhibit the anaphase-promoting complex and localizes to kinetochores in a Bub1p-, Bub3p-, and Mph1p-dependent manner. Mol Cell Biol *22*, 2728-2742.

Xue, Y., Haas, S.A., Brino, L., Gusnanto, A., Reimers, M., Talibi, D., Vingron, M., Ekwall, K. and Wright, A.P. (2004) A DNA microarray for fission yeast: minimal changes in global gene expression after temperature shift. Yeast, 21, 25-39.
